# Retinoic acid-independent expression of *Meis2* during autopod patterning in the developing bat and mouse limb

**DOI:** 10.1186/s13227-015-0001-y

**Published:** 2015-03-14

**Authors:** Mandy K Mason, Dorit Hockman, Lyle Curry, Thomas J Cunningham, Gregg Duester, Malcolm Logan, David S Jacobs, Nicola Illing

**Affiliations:** Department of Molecular and Cell Biology, University of Cape Town, Rondebosch, 7701 South Africa; Sanford-Burnham Medical Research Institute, Development, Aging, and Regeneration Program, La Jolla, 92037 California USA; Randall Division, King’s College London, London, SE1 1UL UK; Department of Biological Sciences, University of Cape Town, Rondebosch, 7701 South Africa; Present address: Weatherall Institute of Molecular Medicine, University of Oxford, Oxford, OX3 9DS UK

**Keywords:** *Meis2*, Retinoic acid signalling, Limb development, Bat wing, Evo-devo, Interdigital webbing

## Abstract

**Background:**

The bat has strikingly divergent forelimbs (long digits supporting wing membranes) and hindlimbs (short, typically free digits) due to the distinct requirements of both aerial and terrestrial locomotion. During embryonic development, the morphology of the bat forelimb deviates dramatically from the mouse and chick, offering an alternative paradigm for identifying genes that play an important role in limb patterning.

**Results:**

Using transcriptome analysis of developing Natal long-fingered bat (*Miniopterus natalensis*) fore- and hindlimbs, we demonstrate that the transcription factor *Meis2* has a significantly higher expression in bat forelimb autopods compared to hindlimbs. Validation by reverse transcriptase and quantitative polymerase chain reaction (RT-qPCR) and whole mount *in situ* hybridisation shows that *Meis2*, conventionally known as a marker of the early proximal limb bud, is upregulated in the bat forelimb autopod from CS16. *Meis2* expression is localised to the expanding interdigital webbing and the membranes linking the wing to the hindlimb and tail. In mice, *Meis2* is also expressed in the interdigital region prior to tissue regression. This interdigital *Meis2* expression is not activated by retinoic acid (RA) signalling as it is present in the retained interdigital tissue of *Rdh10*^*trex/trex*^ mice, which lack RA. Additionally, genes encoding RA-synthesising enzymes, *Rdh10* and *Aldh1a2*, and the RA nuclear receptor *Rarβ* are robustly expressed in bat fore- and hindlimb interdigital tissues indicating that the mechanism that retains interdigital tissue in bats also occurs independently of RA signalling.

**Conclusions:**

Mammalian interdigital *Meis2* expression, and upregulation in the interdigital webbing of bat wings, suggests an important role for *Meis2* in autopod development. Interdigital *Meis2* expression is RA-independent, and retention of interdigital webbing in bat wings is not due to the suppression of RA-induced cell death. Rather, RA signalling may play a role in the thinning (rather than complete loss) of the interdigital tissue in the bat forelimb, while *Meis2* may interact with other factors during both bat and mouse autopod development to maintain a pool of interdigital cells that contribute to digit patterning and growth.

**Electronic supplementary material:**

The online version of this article (doi:10.1186/s13227-015-0001-y) contains supplementary material, which is available to authorized users.

## Background

Understanding the function of genes involved in patterning the tetrapod limb has traditionally been based on leads from human congenital abnormalities, studies using mouse genetics and the experimental manipulation of chicken embryos. This has led to the identification of key genes and pathways that pattern the outgrowing limb, including the three amino-acid loop extension (TALE) homeobox transcription factors, *Meis homeobox 1* (*Meis1*) and *Meis homeobox 2* (*Meis2*), that are expressed in the proximal limb bud [1,2].

One paradigm for understanding how limb outgrowth and patterning is regulated is the ‘two-signal model’ based on pharmacological and transplantation studies in the chick. This model proposes that the proximal-distal (P-D) axis is specified by opposing proximal and distal factors, namely retinoic acid (RA) and fibroblast growth factor (FGF) signalling, that activate or repress *Meis1/2* expression, respectively [3-5]. In this model, *Meis2* expression is restricted to the proximal portion of the outgrowing limb bud, where it plays a role in specifying the proximal limb identity and is used as a marker of the stylopod [2,5-7]. Overexpression/ectopic distal expression of *Meis2* in the developing chick limb results in distal limb defects that include limb axis proximalisation, reductions in the length of distal skeletal elements and the persistence of interdigital webbing [1,2]. Ectopic distal expression of *Meis1* in the developing mouse limb leads to a comparable proximalisation of the distal elements, a reduction of the ulna, a delay in distal element ossification and syndactyly [1]. Ectopic expression of *Meis2* throughout the hindlimb (HL) bud, and the posterior half of the forelimb (FL) bud, leads to delayed expression of *homeobox A13* (*Hoxa13*) in the distal limb bud (a marker of the autopod region) and persistent distal expression of *homeobox A11 (Hoxa11*; a marker of the zeugopod region*)* [8]. These data have been used to build a model of limb bud outgrowth where *Meis1* and *Meis2* play a pivotal role in patterning the proximal limb.

Two distinct thresholds of RA signalling are proposed to delimit the three limb regions, with the higher mediating the stylopod to zeugopod transition and the lower (together with a timing mechanism specified by specific histone acetylation signals) mediating the zeugopod to autopod transition [8]. In this model, RA is synthesised in the flank where *retinol dehydrogenase 10* (*Rdh10*) and *retinaldehyde dehydrogenase 2* (*Aldh1a2/Raldh2*) are expressed [9-12]. Diffusion of RA from the flank through the nascent limb bud forms a gradient, reinforced by degradation of RA through the action of a distally expressed *cytochrome P450* RA-degrading enzyme encoded by *Cyp26b1* [3,5]. Although genetic studies in mice have established that FGF signalling from the apical ectodermal ridge (AER) is required for P-D patterning and the distal repression of *Meis1/2* [13], several genetic studies that abrogate RA synthesis cast doubt on the requirement for endogenous RA signalling in P-D patterning or for expression of *Meis1/2* in the proximal limb [14-16].

Genetic studies support a role for RA signalling later in limb development, maintaining the interdigital tissue of the autopod in an undifferentiated state and mediating cell death events [15,17,18], as well as playing a role in tissue specification events at the digit-interdigit junction during digit development [19]. RA is synthesised in the interdigital region through expression of *Rdh10* and *Raldh2* [10], while RA is degraded in the digit region through *Cyp26b1* expression [20]. Together, these genes regionalise the developing autopod into RA-depleted regions (the condensing digits) and RA-rich regions (undifferentiated interdigital tissue). The interdigital mesenchyme is maintained in mice that are deficient in the RA-signalling pathway (*Rdh10*^*trex/trex*^, *Raldh2*^*−/−*^, *RARβ*^*−/−*^*/RARγ*^*−/−*^ and *RARβ*^*+/−*^*/RARγ*^*−/−*^ mice) suggesting that RA synthesis and subsequent signalling is necessary for the regression of interdigital tissues [15,17-19].

Interdigital tissue is also naturally retained during development in the autopods of several vertebrates, a feature that possibly evolved as an adaptive trait to facilitate locomotion and predation. The bat wing is a clear example of this, containing asymmetrically elongated digits (II to V) that support an expansive wing membrane used in powered flight. In contrast, the bat’s hindlimb digits are short, clawed and (in most species) free, being adapted for crawling and roosting [21]. This unique contrast in autopod morphology provides a powerful comparative system that can be used to inform current developmental models that are established using conventional systems such as the mouse and the chick [22,23].

Based on leads from a transcriptome analysis of developing bat autopods, we present data showing that *Meis2* is upregulated in the interdigital tissue of mammalian autopods. Furthermore, we find that RA signalling and *Meis2* expression are not coupled in the bat or in the mouse autopod.

## Methods

### Sample collection, ethics and preparation

*Miniopterus natalensis* embryos were collected from wild-caught, pregnant females in September and October of 2006 and 2008 from De Hoop Nature Reserve, Western Cape Province, South Africa (Western Cape Nature Conservation Board permit number: AAA004-00030-0035; University of Cape Town Faculty of Science Animal Experimentation Committee application number: 2006/V4/DJ and 2008/V16/DJ), staged and stored as previously described [24,25]. For the microarray experiment, four biological repeats of each developmental stage (Carollia stage 16 (CS16) and Carollia stage 17 (CS17)) were examined. For the reverse transcriptase and quantitative polymerase chain reaction (RT-qPCR) experiment, three biological repeats were examined and the developmental window was extended to include a younger (Carollia stage 15 (CS15)) and an older (Carollia stage 18 (CS18)) stage (Table 1).

**Table 1:** **Fold change data for** ***Meis2*** **transcripts over both the** ***5′-Meis2*** **and the** ***3′-Meis2*** **region**

**Transcript region**	**Experiment**	**Bat (CS17) vs mouse (E13.5)**	**FL vs HL**			
		**FL**	**CS15**	**CS16**	**CS17**	**CS18**
5′-*Meis2* (M400017713)	Microarray	9.9 (8.4 to 11.7)	NA	2 (1.5 to 3.3)	6.1 (5.4 to 12.6)	NA
	qPCR	287.1 (56.5 to 829.9)	0.5 (0.5 to 0.7)	3.0 (2.3 to 3.9)	8.4 (7.7 to 8.5)	7.9 (5.8 to 8.4)
3′-*Meis2* M400000987	Microarray	1.5 (1.2 to 2.3)	NA	1 (0.6 to 1.2)	1.4 (1.2 to 2.6)	NA
	qPCR	8.6 (8.3 to 12.3)	0.9 (0.6 to 1.2)	1.9 (1.6 to 2.6)	5.9 (5.5 to 6.0)	4.9 (2.2 to 5.1)

Median fold changes of biological repeats are given with minimum and maximum fold changes in parentheses. Four biological repeats were tested in the microarray experiments and three in the reverse transcriptase and quantitative polymerase chain reaction (RT-qPCR) experiments. FL, forelimb; HL, hindlimb; NA, not applicable.

Autopods from the FLs and HLs of bat embryos and the FLs of E13.5 mouse ICR embryos (UCT strain 1, University of Cape Town Medical School, Animal Ethics Committee application number: 006/040) were dissected off while immersed in RNA*later*® (Sigma-Aldrich, St Louis, MO, US). This area was distinguished as the dorso-ventrally flattened tissue on the distal portion of the limb and was dissected along the point of constriction of the presumptive wrist or ankle, excluding the tissue associated with the stylopod and the zeugopod as well as that associated with the proto- and plagiopatagium. This autopod tissue is subsequently referred to as either the FL or the HL. Autopod RNA was extracted using the RNeasy® Lipid Tissue Mini Kit (QIAGEN, Hilden, DE) and checked for quality by the Centre for Proteomic and Genomic Research (CPGR, Cape Town, ZA) using the Agilent 2100 Bioanalyser (Agilent Technologies), in addition to running denaturing agarose gels and quantified using the Nanodrop 1000 (Thermo Scientific, Pittsburgh PA, US).

*In situ* hybridisation experiments were performed using several mouse strains. *Meis2 in situ* hybridisation experiments were performed on wildtype NIMR:Parkes mice. RA-signalling pathway *in situ* hybridisation experiments were performed on wildtype C57BL/6 (UCT strain 3) mice (UCT Medical School Animal Ethics number 012/052). The comparison of *Meis2* expression in wildtype (Black Swiss) compared to *Rdh10*^*trex/trex*^ mutant mice, a strain that lacks RA signalling in limbs and exhibits retained interdigital webbing in the hindlimb [9,15], conformed to the regulatory standards adopted by the Animal Research Committee at the Sanford-Burnham Medical Research Institute.

### Microarray analysis

Input RNA samples (0.5 μg) were amplified and labelled using the Amino Allyl MessageAmp™ II Cy3 aRNA Amplification Kit (Ambion, Austin, TX, US). Each labelled amplified RNA (aRNA) sample (250 ng) was hybridised to OpArray™ Mouse v4.0 slides and processed according to the manufacturer’s instructions (OPERON Biotechnologies, Inc., Huntsville, AL, US). Slides were scanned using a Genepix® 4000A scanner (Axon Instruments Inc., Molecular Devices, Sunnyvale, CA, US). Data were captured, background signal calculated and spot quality checked using GenePix™ Pro ver. 6.0 microarray analysis software (Amersham Biosciences, Piscataway, NJ, US). Background correction, normalisation and subsequent pre-processing steps were performed in R ver. 2.6.0 (R Development Core Team). Poor quality features were removed, missing data were imputed, batch artefacts were corrected for and variance filtering was performed. Filtered and pre-processed data were analysed for differential expression using the DEDS package (ver. 1.12). Genes were ranked according to their cumulative *q* value and determined to be differentially expressed if fold change (FC) > 2 (interspecies analysis) or FC > 1.5 (intraspecies analysis) and *q* < 0.01. Single-channel normalised data were used to graph array signal of developmental genes of interest. Data were imported into Microsoft Excel (Microsoft Office 2007), and biological replicate averages were compared using the moderated t-statistic with Benjamini and Hochberg False Discovery Rate correction. Protocols and data were deposited in the NCBI Gene Expression Omnibus [26,27] and can be accessed through the GEO Series accession number GSE51042 (http://www.ncbi.nlm.nih.gov/geo/query/acc.cgi?acc=GSE51042).

### Analysis of *Miniopterus schreibersii* RNA-seq dataset

mRNA-seq datasets (Accession GSE50699) from embryonic autopods of the common bent-wing bat (*Miniopterus schreibersii*) were downloaded from the Gene Expression Omnibus (GEO). For each gene of interest, the longest mouse (GRCmm38) coding transcript sequence (cDNA) was obtained (Ensemble Genome Browser, release 75) and used to perform a BLASTn on the RNA-seq bat sequence dataset. *M. schreibersii* sequences with high similarity were noted [see Additional file 1: Table S2], and the normalised read counts for these were extracted. Where there was more than one high-similarity sequence for a gene of interest, the sum of the normalised read counts was taken.

### 5′ and 3′ RACE and high-fidelity PCR

5′ and 3′ Rapid Amplification of cDNA Ends (RACE)-ready complementary DNA (cDNA) was synthesised from the total RNA extracted from the FLs and heads of CS17 *M. natalensis* and E13.5 *Mus musculus* embryos using the SMARTer™ cDNA Amplification kit (Clontech, Mountain View, CA, US), using oligo(dT) to prime first strand synthesis. Various gene-specific primers (GSPs) and nested gene-specific primers (NGSPs) were designed to generate full-length 5′ and 3′ RACE cDNA transcripts from the *Meis2* locus [Additional file 1: Table S3 and Additional file 2: Figure S1] in combination with Clontech universal primers, using PCR conditions summarised in Additional file 1: Table S4 and Additional file 3. Additional details of PCR conditions, cloning and sequencing can be found in Additional file 3. A *Meis2* overlap region, between the genome-encoded first adenine-rich region and the start of the coding region [see Additional file 2: Figure S1], was amplified from bat (CS17) and mouse (E13.5) embryonic head cDNA as described in Additional file 3.

### Relative qPCR analysis

RT-qPCR was performed following the Minimum Information for Publication of Quantitative Real-Time PCR Experiments (MIQE) conventions [28]. Primers were designed to amplify the transcript regions that were specifically hybridised by the corresponding mouse OPERON probes [see Additional file 1: Table S5]. Experiments were performed using Amino allyl aRNA samples that were reverse transcribed into cDNA using Superscript III Reverse transcriptase (Roche Molecular Diagnostics, Pleasanton, CA, US) and second-round primers (Ambion, Austin, TX, USA). Relative qPCR was performed using the Sensimix SYBR Kit (BIOLINE, London, GB). A portion of each cDNA sample was pooled and serially diluted by a factor of two to generate five standard curve samples for each run. Experiments were performed on the Rotogene 6000 (QIAGEN). Standard curves and E values were generated using the Rotogene 6000 real time rotary analyser software (Ver. 1.7, QIAGEN). Standard curves were plotted for each experiment and the E values [see Additional file 1: Table S5] and Ct values were imported into Microsoft Office Excel 2010 (Microsoft Corporation) and analysed. Outliers that could be attributed to a failed amplification were discarded and the technical repeats averaged. Data were analysed using the efficiency correction method [29]: data were calibrated to the average dilution in the serial dilution standard curve and normalised to the reference gene, *Tbpl1*. After calibration, data were scaled to the bat CS15 HL. Statistical analyses were performed in IBM® SPSS Statistics ver. 22 (IBM Corporation, Armonk, NY, US).

### Whole mount *in situ* hybridisation (WISH)

Bat-specific whole mount *in situ* hybridisation (WISH) probe templates were generated from bat FL pooled cDNA (CS15 to CS18). Primers for *Meis2* WISH probes were designed to include the regions that were specifically hybridised by the corresponding mouse OPERON probes. Primer and probe template information for all bat WISH experiments can be found in the supplementary information [see Additional file 1: Table S6]. PCR products were purified (Promega, Fitchberg, WI, US) and cloned into the pGEM®-T Easy vector (Promega) and sequenced (Central Analytical Facilities, Stellenbosch, WC, ZA) [see Additional file 1: Table S6]. A mouse 5′-*Meis2* probe was generated from RIKEN FANTOM™ mouse clone A830011L22 (GenBank: AK043601.1) (Source Bioscience Lifesciences, Cambridge, GB). Information for mouse probes of RA-signalling-associated genes can be found in previously published work: *mRdh10* [9], *mAldh1a2* [12], *mCyp26b1* [30] and *mRarβ* [31]. Plasmids were linearized using the appropriate restriction enzymes, and DIG-labelled probes were generated using *in vitro* transcription with T3, T7 or SP6 polymerase as required (Roche Molecular Diagnostics). Bat embryos were halved along their rostral-caudal axis prior to WISH to conserve samples and enable direct comparisons between genes from the same developmental stage. WISH was performed as previously described [32] with the following modifications: proteinase K digestion times were as recommended by [33], overnight hybridisation temperature set at 70°C and detection performed using nitro-blue tetrazolium and 5-bromo-4-chloro-3′-indolyphosphate solution (NBT-BCIP).

### Data deposition footnote

NCBI Gene Expression Omnibus GEO Series accession number GSE51042 (http://www.ncbi.nlm.nih.gov/geo/query/acc.cgi?acc=GSE51042). Genbank Accession numbers for all sequences in this paper are given in Additional file 1: Table S7 and S9 to S12.

## Results

### Cross-species microarray analysis reveals a set of genes that are robustly differentially expressed between the bat fore- and hindlimb, including *Meis2* and *Hoxd11*

**Figure 1:**
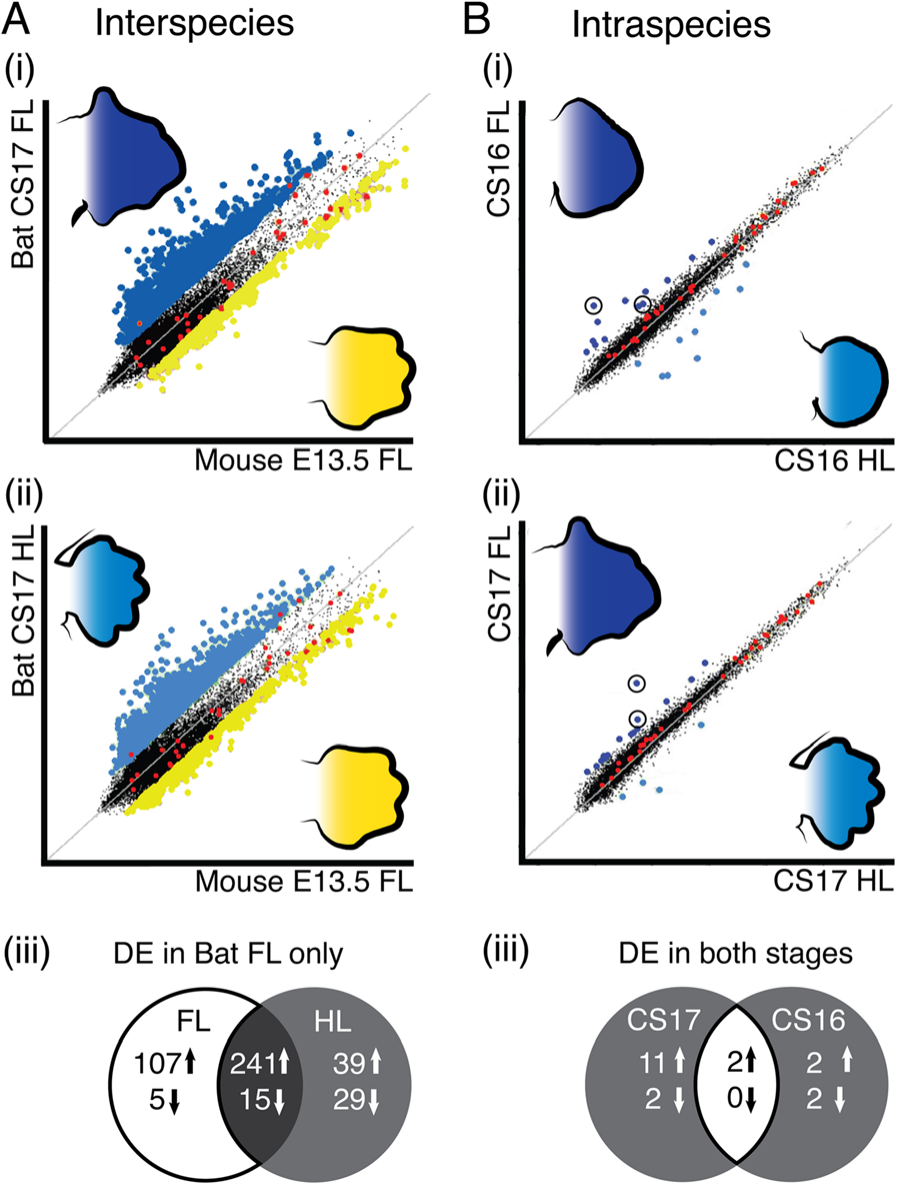
**A microarray comparison of bat forelimb and hindlimb autopod transcript abundance with the mouse forelimb. (A)** Two direct analyses between the array signal from (i) CS17 bat (blue) forelimb (FL) and the mouse (yellow) FL and (ii) CS17 bat hindlimb (HL) and the mouse FL revealed many genes that were significantly differentially expressed (DE). (iii) Commonly DE genes were removed from these lists to identify 112 genes that were uniquely DE between the CS17 FL and the mouse FL. **(B)** Two indirect analyses comparing the bat FL and HL at stages (i) CS16 and (ii) CS17 revealed few differences between the array signal of these limb types. *Hoxd11* and *Meis2* (circled) were identified as highly DE in these comparisons. (iii) Commonly DE genes were found by comparing gene lists using a fold change (FC) filter of either FC > 2 (interspecies) or FC > 1.5 (intraspecies) and a cut-off value of *q* < 0.01.

To identify genes involved in interdigital webbing retention and digit elongation, we performed a cross-species microarray on FL and HL autopods from *Carollia* stage (CS) 16 and CS17 embryos of the Natal long-fingered bat, *M. natalensis*. Mouse FL autopods (E13.5) were used as a reference sample facilitating a three-way comparison. We processed and filtered the data to create a small, high-quality expression data set of five 200 probes (14% of original array) for further analysis. We compared the CS17 bat FL and HL to the mouse E13.5 FL (interspecies comparison) to find genes that were differentially expressed in the bat FL relative to the mouse FL at this equivalent developmental stage [24,25]. A large number of genes were differentially expressed (DE) in this comparison (Figure 1A (i) and (ii)). A comparison of significantly DE genes (FC > 2, *q*-value <0.01) showed that the majority were common to both FL and HL (Figure 1A (iii)). Only 107 upregulated genes and five down-regulated genes were unique to the bat FL analysis and were considered for further analysis.

In contrast to the interspecies analysis, far fewer genes were DE in the intraspecies comparison (Figure 1B (i) and (ii)). Only 15 genes were significantly DE (FC > 1.5, *q* < 0.01) when comparing the bat FL to the HL at CS17 and only six at CS16 (Figure 1B (iii), Additional file 1: Table S7). Two genes were upregulated at both developmental stages and were significantly DE across all comparisons. The most significantly DE gene corresponded to an unannotated probe (M400017713) that mapped upstream of the mouse *Meis2* gene locus, while the second probe mapped to the *homeobox D11* (*Hoxd11*) locus. Here, we explore the role of *Meis2* in autopod formation.

### The microarray probe M400017713 corresponds to the 5′ UTR of the *Meis2* mRNA transcript

BLASTn analysis of the sequence corresponding to M400017713 had a 100% match to a RIKEN mouse clone AK043601, annotated as a cDNA from a RIKEN, full-length enriched, 10-day neonate cortex library. This small (472bp) cDNA clone ended in a polyadenylated tract, and mapped upstream of the mouse *Meis2* locus in the sense direction. The cDNA was predicted to not encode a protein and we consequently refer to it as a long non-coding *Meis* transcript (*lncMeis2*).

**Figure 2:**
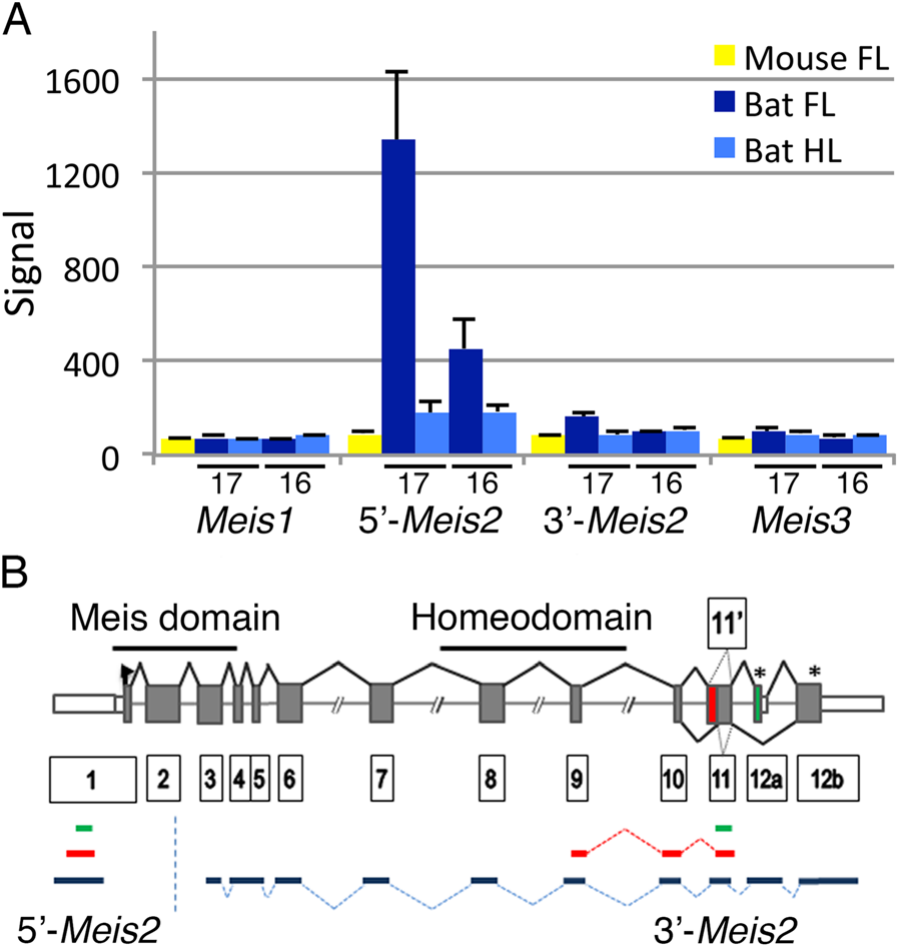
***Meis2*** **expression is upregulated in the bat forelimb as compared to the hindlimb. (A)** A probe that mapped to a region 5′ of the *Meis2* loci (5′-*Meis2*) showed the highest DE in the microarray analysis, with overexpression in the bat CS17 forelimb (FL) in comparison to the hindlimb (HL) and mouse FL. The probe specific to *Meis2* transcripts showed a similar profile of expression but had a lower signal on the array. **(B)** A schematic diagram indicating the conserved intron/exon structure of *Meis2* mRNA transcripts, containing 13 exons (boxed numbers). The regions encoding the Meis domain and homeodomain are indicated. The binding regions of the microarray probes (in green) are shown for *5′-Meis2* and *3′-Meis2*. The position of WISH probes corresponding to *5′-Meis2* and *3′-Meis2* are given in blue, while the targets of the reverse transcriptase and quantitative polymerase chain reaction (RT-qPCR) amplification are shown in red. The alternative spliced exons 11′ and 11, and 12a and 12b are shown in red and green, respectively. The asterisk (*) indicates stop codons and the white boxes represent the untranslated regions (UTRs).

We interrogated FANTOM5 and Genbank databases and performed 5′ and 3′ RACE to validate the presence of this *lncMeis2* RNA transcript. Our analysis [see Additional file 4] reveals that the presence of *lncMeis2* cDNAs in the Genbank databases is a consequence of artificial oligo (dT) priming of internal adenine-rich regions. Our 5′ and 3′ RACE data combined with the FANTOM5 datasets confirm that transcription of the human, mouse and bat *Meis2* transcripts initiates at the same start sites [see Additional file 5: Figure S2]. These analyses indicate that the M400017713 operon probe recognises the 5′ untranslated region (UTR) of the *Meis2* transcript, rather than detecting the presence of a long non-coding RNA transcript (Figure 2). We refer to the M400017713 Operon probe as the 5′-*Meis2* microarray probe henceforth, rather than *lncMeis2*. The microarray probe (M400000987) annotated as *Meis2* mapped to a more 3′ region, within exon 11 (Figure 2), which we refer to as the 3′-*Meis2* microarray probe.

### *Meis2* transcripts are upregulated in developing bat wings

The microarray signal from the 5′-*Meis2* probe was compared alongside that from the 3′-*Meis2* probe, in addition to the signal from the *Meis1* and *Meis3* probes (Figure 2A). The signal from the 5′-*Meis2* probe was higher in the FL of both the CS17 (over sixfold) and CS16 (twofold) bat (Table 1) compared to their HLs and was significantly higher (over ninefold) in the CS17 FL compared to that of the mouse (Table 1, Figure 2A). The 3′-*Meis2* probe was also higher in CS17 FL as compared to the HL (1.4-fold) and the mouse FL (1.5-fold), but these fold changes were modest with the probe having a lower array signal overall. Both mouse probes have high similarity to the bat genomic sequence (96% and 98%, respectively). The array signals from the *Meis1* and *Meis3* probes were low and not significantly different between limb types (Figure 2A). These data correspond with recently published RNA-seq data in the closely related bat species *M. schreibersii* [see Additional file 6: Figure S3] [34,35]. Our analysis of the *M. schreibersii* dataset showed that *Meis2* was the most highly expressed of the three *Meis* genes. Its expression in tissue taken from the anterior and posterior regions of the early limb bud (CS14) corresponds well to the known expression pattern of *Meis2* at early stages. In the pooled CS15-17 bat limbs, *Meis1 and Meis3* have a very low read count in both limb types. In contrast to this, *Meis2* transcripts are abundant in the FL interdigital tissue (digits II to V), while expression is low in FL digit I, FL digits II to V and HL samples [see Additional file 6: Figure S3] [34].

### RT-qPCR validation confirms the upregulation of *Meis2* transcripts in autopods of developing bat forelimbs

**Figure 3:**
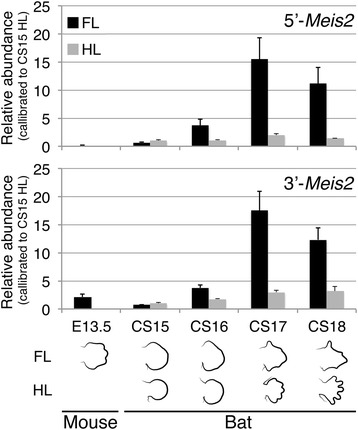
**RT-qPCR products corresponding to** ***Meis2*** **transcripts are elevated in the bat forelimb compared to hindlimb autopods.** The abundance of *Meis2* transcripts was quantified by reverse transcriptase and quantitative polymerase chain reaction (RT-qPCR) in the E13.5 mouse forelimb autopods and in bat forelimb (FL) and hindlimb (HL) autopods from early stages of autopod patterning (CS15) to late stages of autopod differentiation and growth (CS18), using primers designed to either the 5′ region of *Meis2* (*5′-Meis2*) or to the 3′ region of *Meis2* (*3′-Meis2*). *Meis2* transcript abundance was higher in the bat CS17 FL compared to the mouse E13.5 FL, for both primer sets. The transcript abundances of the FL samples were over twofold higher than that of HL from CS16 onwards, with abundance peaking in the CS17 FL. Statistical analyses of these data can be found in the supplementary results.

We validated the array signal of mRNA transcripts hybridising to either the 5′- or 3′-*Meis2* probes by reverse transcriptase (RT)-qPCR analysis using primers designed to amplify each probe-binding region (Figure 2B). The 5′-*Meis2* and 3′-*Meis2* RT-qPCR reactions gave similar results for CS15 through to CS18 (Table 1, Figure 3). Expression was low in both the CS15 FL and HL. In the FL, expression increased at CS16, peaked at CS17 and dropped slightly at CS18. HL expression remained low throughout development (Figure 3). A large difference between the FL and HL abundance (over fivefold) is seen at CS17 for both probes with moderate differences seen at CS16 (Table 1). These results validate the microarray data and support the finding that *Meis2* is overexpressed in bat FL from developmental stage CS16 onwards as compared to the HL.

### Expression of *Meis2* occurs in the interdigits of the mouse autopod and the interdigital webbing of the bat forelimb but is absent from the bat hindlimb

**Figure 4:**
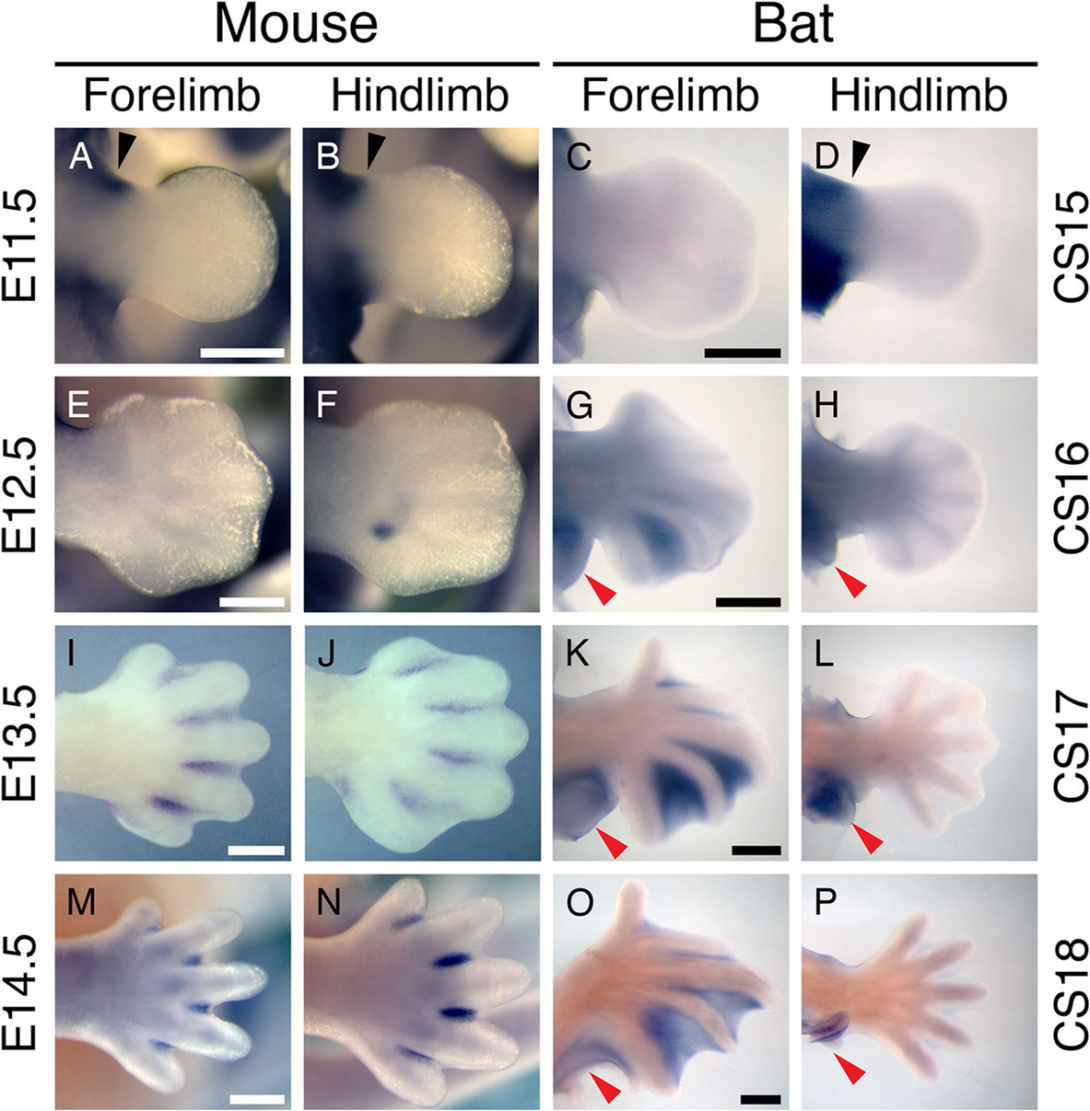
***Meis2*** **transcripts are detectable in the distal autopod domain in mouse and bat limbs.** During early limb development in the mouse, the 3′-*Meis2* WISH probe detects expression in the proximal region of the limb bud (black arrow, **A, B**). A similar pattern can be seen in equivalently staged bat limbs (black arrow, **C**, **D**) and is comparable between forelimbs (FLs) and hindlimbs (HLs). During the early stage of mouse autopod development, *Meis2* is not seen in the FL **(E)**, but a small, comma-shaped domain of expression can be found in the HL **(F)** with this probe. In contrast to this, expression is found in the region between digits IV and V of the bat FL **(G)** and is absent in the HL **(H)**. At later stages of digit formation, *Meis2* can be seen in all of the interdigital regions of both the FL **(I)** and the HL **(J)** of the mouse and the FL of the bat **(K)** but is still absent from the bat HL **(L)**. In the mouse, expression is restricted to the proximal region of the regressing interdigital mesenchyme (**I, J, M** and **N**) but is maintained in the webbing of the bat FL **(O)** while a very faint stripe of expression can be seen in the HL interdigital region **(P)**. It is notable that *Meis2* expression can be found in the FL and HL patagia throughout their development (red arrow in **G, H, K, L, O, P**). Dorsal views of autopods are shown. Scale bars represent 500 μm.

We characterised the expression pattern of *Meis2* in autopods by WISH over sequential stages of autopod development in both mouse (E11.5 to E14.5) and bat (CS15 to CS18) embryos, using WISH probes derived from either the 5′ UTR (*5′-Meis2*) or the *Meis2*-coding region (*3′-Meis2*) (Figure 2B). In the mouse, we confirmed that *Meis2* is expressed in the proximal limb bud region at early stages of limb bud outgrowth (E11.5) (Figure 4A, B). The earliest indication of *Meis2* expression in the distal region of the mouse limb is found in the E12.5 HL, as a distinct comma-shaped region proximal to the presumptive digit rays IV and V (Figure 4F and Additional file 7: Figure S4) (13/15 embryos using the 3′-*Meis2 in situ* probe). This region of expression is not observed in the FL (Figure 4E) and is not evident in later stages of HL development (Figure 4J, N). *Meis2* expression in the interdigital tissue is first seen as a very faint stripe of staining in the E13 FL when viewed frontally [see Additional file 8: Figure S5A]. Expression is clearly visible in the proximal interdigital region of FL and HL autopods in E13.5 embryos (Figure 4I, J, Additional file 8: Figure S5C and J) and is absent from the most distal regions. The restriction of *Meis2* expression to the proximal interdigital tissue of the HL is clearly visible in the E14.5 HL (Figure 4N, Additional file 8: Figure S5J to L).

The early expression of *Meis2* in the CS15 bat is comparable to that of the E11.5 mouse, with expression confined to the proximal region of the limb bud (Figure 4C, D). The first evidence of expression of *Meis2* in the bat autopod is in the CS16 FL where a strong domain of expression can be seen in the interdigital region between digits IV and V (Figure 4G). A less intense region of staining is apparent in the interdigital region between digits III and IV, and no expression is evident in interdigital regions I to II or II to III at this stage (Figure 4G). Interestingly, *Meis2* is also strongly expressed in the presumptive plagiopatagium and uropatagium from CS15 onwards (red arrows in Figure 4G, H, K, L, O, P). At later stages, *Meis2* expression is evident in all interdigital tissues of the FL, with a strong proximal bias (Figure 4K, O). The ‘comma-shaped’ domain of expression observed in the mouse HL is not found in the HL of any bat embryos (Figure 4H and Additional file 9: Figure S6). Although *Meis2* expression is undetectable in CS15 to CS17 HLs, we could detect a faint line interdigital expression in CS18 HL autopods (Figures 4P).

WISH using the 5′- and 3′-*Meis2* probes gave similar results, except in mouse HLs where only the 3′-probe detected the aforementioned ‘comma-shaped’ domain [see Additional file 9: Figure S6 and Additional file 10: Figure S7]. This could be due to variation in the sensitivity of the different-sized probes or a difference among the 5′ UTR of *Meis2* transcripts for this domain.

### *Meis2* transcripts are expressed in the absence of RA signalling in the interdigital tissue of *Rdh10* mutants

Our discovery of *Meis2* expression in the interdigital tissue of the developing mouse and bat gave us the opportunity to test the dependence of its expression on RA signalling and examine the role of RA signalling in shaping the bat FL autopod with its retained interdigital membranes.

**Figure 5:**
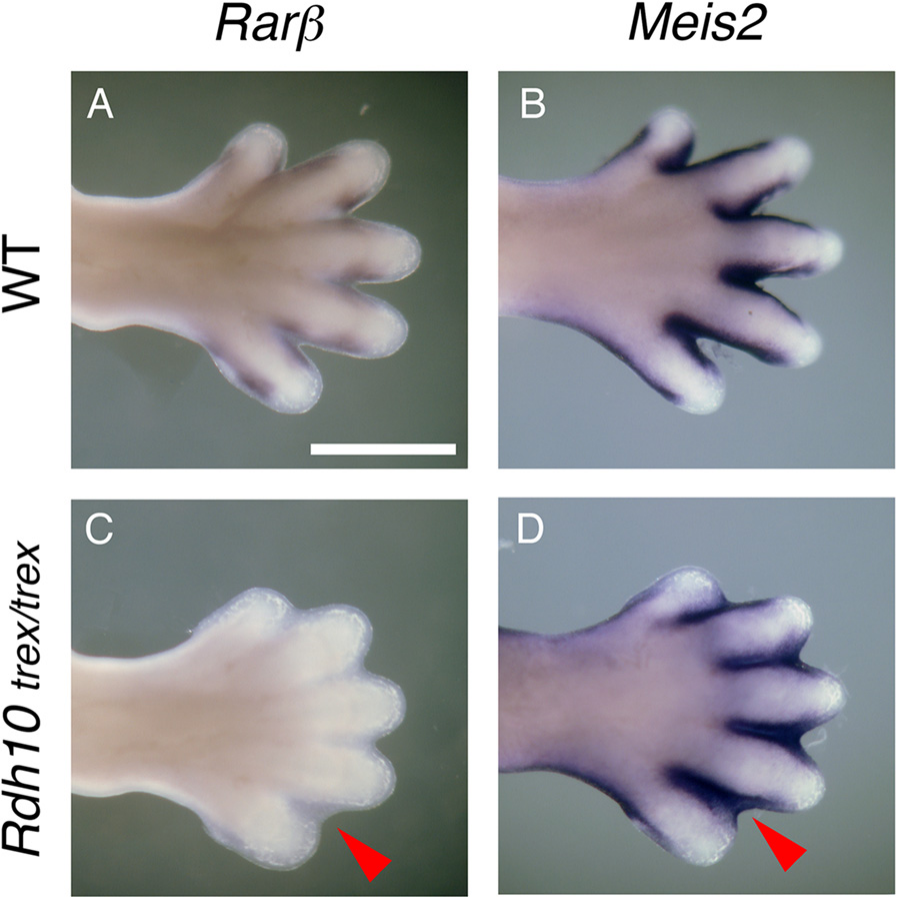
***Meis2*** **is expressed independently of RA signalling in the interdigital tissue of the mouse.** In the wildtype (WT) E14.5 mouse, *Rarβ* and *Meis2* expression can be seen in the intra-digit junction **(A)** and tissue surrounding the digits **(B)** of the HL, respectively. RA signalling is absent in the mouse *Rdh10* ^*trex/trex*^ mutant as evidenced by the lack of *Rarβ* expression in the retained interdigital webbing of the HL **(C)**. *Meis2* is still expressed in this region **(D)**, indicating that its expression is not dependent on RA signalling. Dorsal views of autopods are shown. Scale bar represent 500 μm.

We first examined the expression of *Meis2* and *Rarβ* in the limbs of the *Rdh10*^*trex/trex*^ mouse. *Rarβ* expression is directly induced by RA [36] and plays a role in RA-induced interdigital tissue loss [17,18]. In E14.5 mouse embryos, *Rarβ* is expressed at the digit-interdigit junction in wildtype embryos but is missing in *Rdh10*^*trex/trex*^ mutants (Figure 5A, C), demonstrating the absence of active limb RA signalling in *Rdh10*^*trex/trex*^ mutants. Despite the lack of RA signalling, *Meis2* is still strongly expressed throughout the retained interdigital tissue of the *Rdh10* mutant hindlimb autopod indicating that *Meis2* expression does not rely on RA signalling in this domain (Figure 5B, D).

### Retention of interdigital webbing in the bat forelimb is not a consequence of diminished RA signalling

In mice, interdigital RA signalling plays a role in apoptotic events that result in freely separated digits [15,19,37]. We used a combination of our microarray expression dataset and *in situ* hybridisation studies to investigate whether retention of the interdigital membrane in the bat FL autopod was linked to downregulation of RA signalling in comparison to the bat HL and mouse FL.

An analysis of the microarray data shows that there is no significant difference in the abundance of mRNA transcripts for the key RA-signalling genes between bat FL and HL autopods [see Additional file 10: Figure S7]. The *Rdh10*, *Aldh1a2* and *Cyp26b1* genes, which encode enzymes that perform the entirety of RA synthesis and degradation in the mouse limb [9,11,15,20], have signals that are similar among all limb types. *Rarβ* plays a predominant role in limb RA signal transduction in the interdigital region and also showed very little signal differences across limb types [see Additional file 10: Figure S7A].

**Figure 6:**
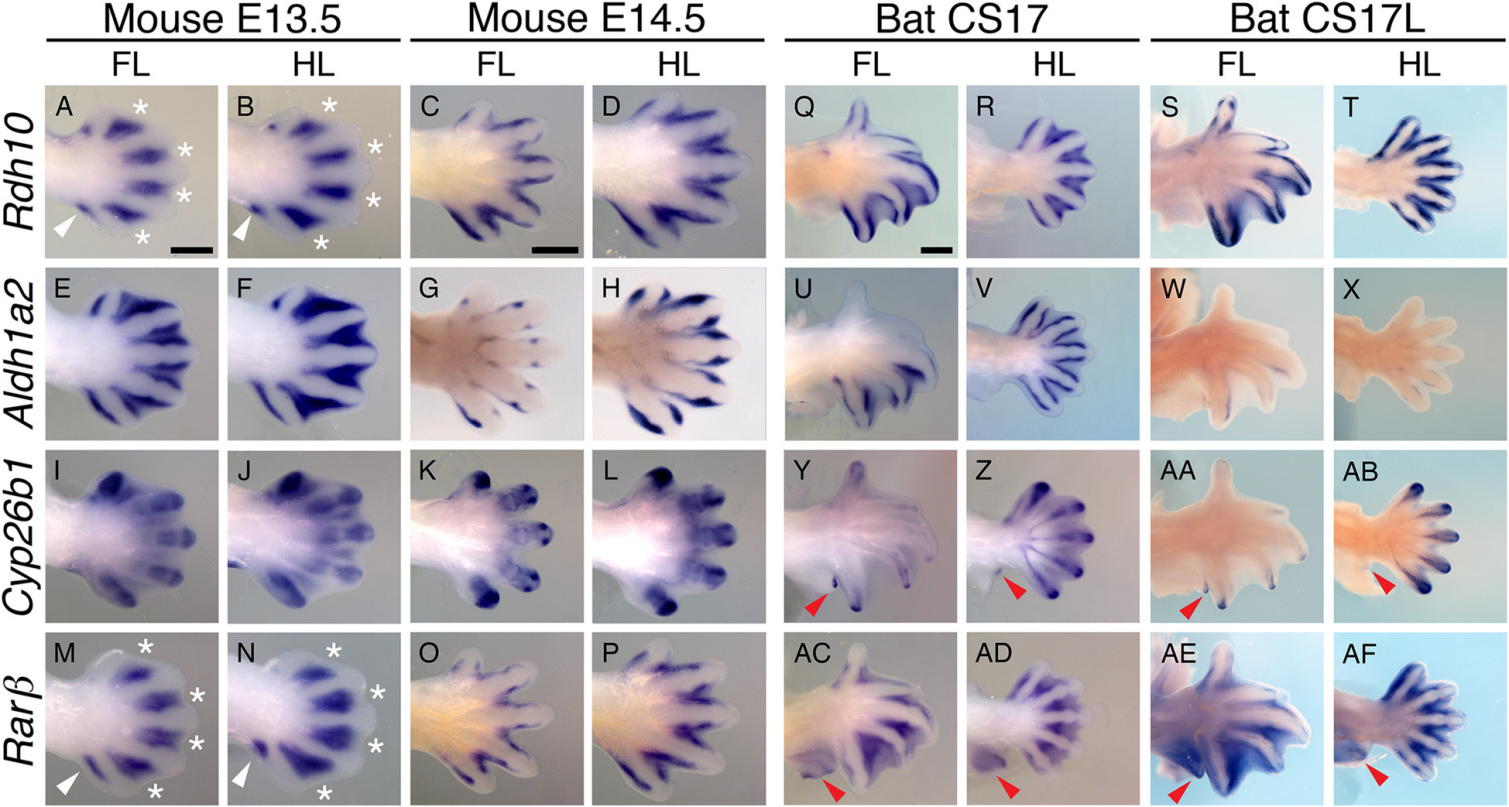
**RA signalling is active in the interdigital region of bat forelimb and hindlimb autopods.** Expression of genes involved in retinoic acid (RA) synthesis, degradation and signalling in the bat CS17 and CS17L as compared to those of the E13.5 and E14.5 mouse indicate that this pathway is active in both the FL and the HL interdigital tissue of these mammals. *Rarβ* and *Rdh10* expression in the mouse limbs **(A, B, C, D** and **M, N, O, P)** is not visible in the distal interdigital region (white asterisk) and is concentrated in the proximal posterior necrotic zone (white arrows). In bat limbs, *Rdh10* **(Q, R, S, T)** and *Aldh1a2* **(U, V, W, X)** are expressed in the region bordering the developing digits, as well as being weakly expressed in the interdigital tissue. In contrast, *Aldh1a2* is expressed in the interdigital tissue with a distal concentration gradient in mouse FL and HL at E13.5 **(E, F)**. This expression is restricted to tissue flanking the distal digit rays by E14.5 **(G, H)**. In both the mouse and the bat limbs, *Cyp26b1* **(I, J, K, L** and **Y, Z, AA, AB)** is expressed in the developing digital rays and is highest at the tips of the digits. In bat, *Cyp26b1* (Y, Z, AA, AB) is also weakly expressed in the interdigital tissue. Red arrows indicate *Cyp26b1* (Y, Z, AA, AB) and *Rarβ* **(AC, AD, AE, AF)** expression in the patagia. Dorsal views of autopods are shown. Scale bars represent 500 μm.

Although these data indicate that the transcript levels for these enzymes are similar in the bat FL and HL, and mouse FL autopods, we performed WISH to examine whether their spatial expression patterns differed during the stages of interdigital regression. Expression of genes involved in RA synthesis, degradation and signalling in the bat CS17 FL and HL as compared to those of the E13.5 mouse confirms that this pathway is active in the FL and the HL interdigital tissue of both mammals. In the mouse FL, expression of RA synthesising genes (*Rdh10*, Figure 6A, B and *Aldh1a2*, Figure 6E, F) is restricted to the interdigital tissue with an absence of *Rdh10* expression at the distal boundary. The RA-degrading enzyme *Cyp26b1* is expressed strongly in the digits (strongest in digit I) and weakly in the interdigital tissue at E13.5 (Figure 6I, J), becoming restricted to the digit tips at E14.5 (Figure 6K, L). *Rarβ* is similar to *Rdh10* and is restricted to the more proximal regions of the interditigal tissue and the posterior necrotic zone adjacent to digit V in E13.5 embryos (Figure 6M, N). In the bat, intense *Rdh10* expression is seen surrounding all the FL and HL digits with more diffuse expression evident in all interdigital regions (Figure 6Q, R). This concentrated *Rdh10* expression, adjacent to the digit rays, can be seen in later stages of development in mouse autopods (Figure 6C, D). *Aldh1a2* follows this pattern in the HL (Figure 6V); it is notable that *Aldh1a2* expression is absent in the regions adjacent to digits I and II of the FL (Figure 6U). This agrees with our analysis of the Wang *et al.* [34] RNA-seq data which showed threefold lower expression of *Aldh1a2* in digit I (FI) compared to digits II to V (FD) and sixfold lower expression in interdigital I to II (FW) compared to interdigital II to V (FF) in pooled CS15-CS17 FL *M. schreibersii* autopods [see Additional file 11: Figure S8B]. *Cyp26b1* is expressed in the tips of the FL digits with the weakest expression in digits II and III (Figure 6Y, AA). *Cyp26b1* is also expressed in the interdigital tissue and the extending tip of the plagiopatagium alongside digit V of the bat FL (Figure 6Y, AA). The expression of *Cyp26b1* in the bat HL appears more prominent and is strongest in the tendons and digit tips, with diffuse expression in the regressing interdigital tissue (Figure 6Z, AB). Expression of *Cyp26b1* is also evident in the uropatagium adjacent to digit V (Figure 6Z, AB). *Rarβ* is expressed throughout the interdigital tissue of both the bat FL and HL (Figure 6AC, AD), with stronger proximal expression. It is interesting to note that *Rarβ* is also expressed in the plagio- and uropatagium, in a broader domain than *Cyp26b1* (red arrow in Figure 6AC, AD, AE, AF).

## Discussion

In this paper, we describe a novel, RA-independent expression domain for the transcription factor *Meis2* in interdigital tissue during mouse and bat embryonic limb development. The expression of *Meis1/2* in the proximal limb during early limb bud outgrowth has been described in several vertebrates including zebrafish, urodele, chick and mouse embryos [1,2,38-44]. Consistent with these reports, we show that early *Meis2* expression in the proximal limb mesenchyme of Natal long-fingered bat (*M. natalensis)* embryos appears to be conserved, resembling that of the mouse. Additionally, we show that *Meis2* has a second phase of expression in the autopod later in limb development. *Meis2* expression is robustly upregulated in *M. natalensis* CS16 and CS17 FL autopods compared to HL autopods. Furthermore, *Meis2* transcripts are expressed in the interdigital tissue of mouse FL and HL autopods, prior to tissue regression.

*Meis2* is conventionally described in terms of its role in specifying the proximal limb, that is the stylopod [3,6,7]. The proposed dependence of *Meis1* and *Meis2* on RA signalling from the embryonic flank is an important component of this two-signal model of proximal-distal patterning [4,5]. However, the role of RA signalling in this model has been challenged. Genetic loss-of-function experiments show that expression of *Meis1/2* expression is maintained in the limbs of *Raldh2*^−/−^, *Raldh3*^−/−^ and *Rdh10*^*trex/trex*^ mouse embryos, and even in *Rdh10*^*trex/trex*^ embryos treated with a retinoic acid receptor (RAR) antagonist [14-16]. These mutants show no endogenous limb-field RA signalling as indicated by a *retinoic acid responsive element-β-galactosidase* (*RARE-lacZ*) transgenic reporter, shown to be sensitive to 0.25 nM RA [16]. Although these studies show that RA signalling is required for mouse FL bud initiation, as evidenced by stunted FL buds, RA is not required for HL bud initiation or proximal expression of *Meis1/2* in either the FL or HL [14,16,45]. We show that *Meis2* is expressed in the interdigital membrane of *Rdh10*^*trex/trex*^ mice, despite an absence of RA signalling (clear from the prominent syndactyly defect and lack of RARE-lacZ activation/expression [15]). This observation is consistent with a model of limb patterning in which the expression of *Meis2* does not require RA signalling.

A role for *Meis2* in shaping autopods has not been previously considered; we do so in the context of RA signalling and interdigital apoptosis. RA signalling is known to play an important role in autopod patterning by regulating the expression of genes that activate cell death and inhibit cell differentiation in the interdigital regions, as well as controlling tissue specification events at the digit-interdigit junction [15,19,37]. Mutants that are deficient in RA signalling display syndactyly [46] raising the possibility that diminished RA signalling in the bat FL might explain the retention of the interdigital membrane. We show that this not the case. An analysis of mRNA expression data from microarrays (this study), as well as recently published RNA-seq datasets [34], did not identify any significant differences in the expression of key enzymes in the RA synthesis pathway (*Rdh10*, *Raldh2* and *Cyp26b1*) in bat FL autopods compared to HL autopods*.* Furthermore, *in situ* hybridisation analysis shows that the RA-responsive gene, *Rarβ*, is expressed in the interdigital tissue of bat FL and HL autopods, as well as in the plagiopatagium and uropatagium, suggesting that RA signalling is active in these tissues. Therefore, a loss of RA signalling does not explain the phenotype of retained interdigital webbing phenotype in the bat FL autopod.

It is possible that the bat FL lacks the downstream response to RA signalling required for complete removal of interdigital tissue. Another possibility is that RA signalling in the bat FL is modulated, allowing the thinning but not the complete removal of the bat wing interdigital membranes. RA signalling is known to activate BMP-mediated cell death in the mouse limb interdigital region [37]. However, this appears to be limited in the bat by the expression of *Gremlin* (a BMP antagonist) in the FL at CS16 and CS17, *Msh homeobox 1* (*Msx1*) and *Msh homeobox 2* (*Msx2*) (apoptosis markers) are still expressed [23]. Also, expression of *fibroblast growth factor 8* (*Fgf8*) in the interdigital mesenchyme of the bat CS16 and CS17 FL likely limits distal apoptosis and prevents the ingression of interdigital tissue adjacent to digits II to V. This hypothesis is supported by experiments showing that inhibition of FGF in the bat FL (using a bead soaked in the FGF inhibitor SU5402) alongside the addition of ectopic BMP causes increased cell death in interdigital regions [23]. Thus, while RA-mediated apoptosis is likely occurring in the bat interdigital membranes, this process appears to be modulated by the presence of *Gremlin* and *Fgf8* in the FL, resulting in the thinning of the interdigital tissue rather than the complete regression of the membranes.

In both the mouse and bat autopod, *Meis2* may mark the regions of interdigital tissue that are retained as a proliferating pool of cells that feed digit development. In the mouse, *Meis2* is predominantly expressed in the more proximal interdigital regions, which undergo thinning, but do not appear to regress [47,48]. This observation fits the differential growth model of autopod formation whereby the distal regions of the interdigital tissue undergo massive apoptosis [47-49]. These distal regions are made up of cells that have previously received FGF signals from the AER and subsequently undergo apoptosis due to a loss of this signalling [47-49]. The digit regions, over which FGF signals are maintained, proliferate, extending distally at a faster rate than that of the interdigital regions. *Meis2* may play a role in the survival of the regions of interdigital tissue that are retained during this process.

This proposal is consistent with our observations in the bat. The pattern of *Meis2* expression in the FL autopod (hand plate), and lack of expression in the HL autopod (footplate), is associated with digit and interdigital tissue outgrowth, rather than interdigital regression. *Meis2* expression in the bat hand plate first appears at CS16 between digits IV and V. This region of the hand plate undergoes a rapid expansion at this stage of development [24,25]. In the Carollia stage 16 late (CS16L) hand plate, *Meis2* expression expands to the tissue between digital rays II to V. The interdigital tissue between the thumb and digit II recedes by stage CS17, while the tissue between the digits II and V persists to form the chiropatagium (wing membrane) that extends distally as digits II to V grow out rapidly. These observations extend to other bat species. *Meis2* transcripts are abundantly expressed in the interdigital tissue of FL digits II to V, but not in the HLs for stages CS15L-CS19 in *M. schreibersii* embryos [34,50]. Elevated expression of *Meis2* has also been noted in tissue taken from FL digits and interdigital tissue (II to V) compared to FL digit I or the HL (digits I to V) at the later (fetal) stages in *Myotis ricketti* and *Hipposideros armiger* [51]. Therefore, it appears that the expression of *Meis2* coincides with the thinning and growth of the interdigital tissue into expansive webbing, a process that begins during autopod formation and continues throughout fetal development [25]. *Meis2* expression may mark and determine the fate of this region by keeping these cells in a proliferating, undifferentiated state, similar to the role of *Meis1/2* in retinal progenitor cells [52].

## Conclusions

In the developing bat forelimb, there is a rapid growth and elongation of digits II to V and their associated patagia; these processes contrast greatly with what is occurring in the hindlimb where digits I to V remain shortened and become free. The expression of *Meis2* transcripts in the developing bat hand plate corresponded to these processes and is consistent with an important role for *Meis2* in stimulating both the outgrowth of digits II to V and the expansion of the chiropatagium in the bat. Furthermore, expression of *Meis2* in developing mouse FL and HL autopods may play a similar, albeit less dramatic, part in sculpturing the tetrapod hand and foot, by maintaining a pool of proliferating interdigital cells that stimulates digit outgrowth. We have shown that these functions of *Meis2* in the autopod operate independently of RA signalling.

## Additional files

Additional file 1: Table S1. Summary of experimental samples. The average and standard deviation of measurements for each staging group are given. **Table S2.** We used a BLAST analysis to find the top hit for each gene ID in the Wang *et al.* [34] dataset, using an Ensembl mouse transcript as the query. **Table S3.** Primers used to characterise *Meis2* transcripts. **Table S4.** PCR cycling conditions for 5′ and 3′ RACE. **Table S5.** Summary of RT-qPCR gene targets with accession numbers for transcripts, amplicon sizes and target region, intron size and the efficiencies (E) and standard error of the mean (s.e.m). **Table S6.** Summary of primers used to generate WISH probes Accession numbers or references for probes are given where relevant. **Table S7.** Top differentially expressed genes that were over- or under-expressed in the comparisons. Gene names denoted with a dagger (†) indicate that probes were annotated by Blat analysis. Percentage similarity (%) gives the OPERON probe sequence similarity to that of the bat (*Myotis lucifugus*). Asterisk indicates when comparisons are significantly different (**P* < 0.05, ***P* < 0.01). **Table S8.** Summary of 5′ start site of EST clones that match AK043601, relative to *Homo sapiens* chromosome 15. **Table S9.** Summary of start sites from *lncMeis2* 5′ RACE reactions, mapped to chromosome 15 on the *H. sapiens* GRCH37 assembly. **Table S10.** Summary of termination sites from *lncMeis2* 3′ RACE reactions, mapped to chromosome 2 of the *M. musculus* GRCm38 assembly, contig GL429805 of the *M. lucifugus* Myoluc2.0 assembly and chromosome 15 of the *H. sapiens* GRCH37 assembly. **Table S11.** Summary of start sites of *Meis2* 5′ RACE reactions mapped to contig GL429805 of the *M. lucifugus* Myoluc2.0 assembly, chromosome 2 of the *M. musculus* GRCm38 assembly and chromosome 15 of the *H. sapiens* GRCH37 assembly. **Table S12.** Genbank Accession numbers for the *Meis2* overlap clones. Additional file 2: Figure S1. Summary of the positions of each RACE primer and PCR product relative to the bat *Meis2* locus. The red blocks indicate the different bat EST clone boundaries corresponding to the different 5′ and 3′ RACE and PCR reactions. The green arrows represent the 3′ RACE primers and the blue arrows represent the 5′ RACE primers. The position of polyA tracts on the mRNA and genome template are indicated by black arrows. Primers used in a standard PCR reaction to amplify cDNA synthesised with random hexamers, over these polyA tracts, are shown with yellow arrows. The presence of a polyadenylation-recognition sequence (AAAUAAA), 16 nucleotides from the polyA tail, is shown. The polyA tracts are present in the genome *Meis2* contig and are not preceded by a polyadenylation-recognition sequence. Additional file descriptions text (including details of how to view the file, if it is in a non-standard format). Additional file 3:
**Supplementary Methods.** Additional information on experimental conditions for 5′ and 3′ RACE reactions to map *Meis2* transcripts and high fidelity PCR. Additional file 4:
**Supplementary Results.** Analysis showing that the microarray probe M400017713 corresponds to the 5′ UTR of *Meis2* transcripts and statistical analysis of relative qPCR data for *5′-Meis2* and *3′-Meis2*. Additional file 5: Figure S2. Histogram and screen-shot. Histogram (A) summarises the frequency of the 5′ boundaries of mouse and human ESTs that match *lncMeis2*, relative to 5′ RACE results from mouse and bat limb and head cDNA mapped to the strand of human chromosome chr15:37392600-37392800. A screen-shot (B) summarises the distribution of human and mouse Fantom5 CAGE tags that map to the equivalent region on the Zenbu browser, confirming that this region corresponds to the major TSS for *Meis2*. Additional file 6: Figure S3. RNA-seq reads for *Meis2*. RNA-seq reads from (A) CS14 forelimb anterior, medial and posterior (A, M and P, respectively) and their corresponding hindlimb regions show that the expressions of the *Meis* genes are highest in the hindlimb corresponding to the anterior and posterior limb bud region, with *Meis2* being the most predominantly expressed transcript. The *Meis2* transcript was also the most abundant *Meis* transcript in the (B) pooled CS15-CS17 limb samples that compared expression among digit and interdigit regions of the forelimb (FL) and the hindlimb (HL). The FL tissues were separated into the anterior portion (I) containing either digit I or the adjacent interdigit and the posterior portion (II to V) containing digits II to V or the corresponding interdigits. The highest expression was found in the forelimb posterior interdigits (FL (II to V)), with lowered expression occurring in the digits of FL (I), FL (II to V), interdigits FL (I) and the digits and interdigits of the hindlimb. Data sourced from Wang *et al.* [34]. Additional file 7: Figure S4. The 3′-*Meis in situ* probe in the mouse HL detects a distinct ‘comma-shaped’ domain of expression during early autopod formation. Lateral (A, B), rostral (C) and ventral (D) views of whole E12.5 and E13.0 embryos show the full extent of 3′-*Meis2* probe signal with the distinct footplate signal occurring in both left and right limbs (white arrows A, B and C). A high-magnification image of the footplate at E12.5 and E13.0 shows the full extent of this staining before it is lost at E13.5. Additional file 8: Figure S5. Hybridisation of the 3′-*Meis2* probe in the developing mouse autopod is limited to interdigital tissue that is retained. A time series of the expression in both the forelimb and the hindlimb indicates that *Meis2* is expressed in both the dorsal and ventral surface of the autopod, in the region of the interdigits, over the period of interdigital thinning. A schematic of the staining over these stages gives an indication of the growth of the autopod and the regression of the interdigital tissue during this time. Dorsal views of autopods are shown. Scale bar represents 500 μm. Additional file 9: Figure S6.
*Meis2* expression in developing bat limbs showing the biological repeats that were performed with both the 5′-*Meis2* and the 3′-*Meis2 in situ* probes. Dorsal views of autopods are shown. Scale bars represent 500 μm. Additional file 10: Figure S7.
*Meis2* expression in developing mouse limbs indicates that there are few differences in signal between the *5′*-*Meis2* and the *3′*-*Meis2 in situ* probes. Both probes show proximal expression in the developing FL and HL limb buds at E11.5 (A, B, C, D). The E12.5 autopods do not show any signal for the *5′-Meis2* probe (E, G) but a small comma-shaped domain of expression is seen in the HL with the *3′-Meis2* probe (H). However, during later stages of development (E13.5 and E14.5), both probes detect the same spatial expression pattern. *Meis2* is expressed in the interdigital regions and is maintained in the proximal region of the interdigital tissue as regression occurs. Dorsal views of autopods are shown. Additional file 11: Figure S8. RA synthesis, degradation and signalling data (A) The expression of genes involved in RA synthesis, degradation and signalling were not significantly differentially expressed in microarray analyses comparing E13.5 mouse FL, CS16 and CS17 *M. natalensis* FL and HL. (B) Analysis of RNA-seq data from pooled CS15-CS17 *M. schreibersii* FL and HL samples. The FL tissues were separated into the anterior portion (I) containing either digit I and the adjacent interdigit or the posterior portion (II to V) containing digits II to V and the corresponding interdigits. Data sourced from Wang *et al.* [34].

## Abbreviations

AER: apical ectodermal ridge
*Aldh1a2/Raldh2*: *retinaldehyde dehydrogenase 2*
aRNA: amplified RNA
cDNA: coding transcript sequence
CS15: Carollia stage 15
CS16: Carollia stage 16
CS16L: Carollia stage 16 late
CS17: Carollia stage 17
CS18: Carollia stage 18
*Cyp26b1*: *cytochrome P450* RA-degrading enzyme
DE: differentially expressed
E13.5: mouse embryo 13.5 days
FC: fold change
FGF: fibroblast growth factor
*Fgf8*: *fibroblast growth factor 8*
FL: forelimb
GSP: gene-specific primer
HL: hindlimb
*Hoxa11*: *homeobox A11*
*Hoxa13*: *homeobox A13*
*Hoxd11*: *homeobox D11*
*M. natalensis*: *Miniopterus natalensis*
*M. schreibersii*: *Miniopterus schreibersii*
*Meis1*: *Meis homeobox 1*
*Meis2*: *Meis homeobox 2*
MIQE: Minimum Information for Publication of Quantitative Real-Time PCR Experiments
*Msx1*: *Msh homeobox 1*
*Msx2*: *Msh homeobox 2*
NBT-BCIP: nitro-blue tetrazolium and 5-bromo-4-chloro-3′-indolyphosphate solution
P-D: proximal-distal
RA: retinoic acid
RACE: Rapid Amplification of cDNA Ends
RAR: retinoic acid receptor
*RARE-lacZ*: *retinoic acid responsive element-β-galactosidase*
*RARβ*: *retinoic acid receptor, beta*
*Rdh10*: *retinol dehydrogenase 10*
RT-qPCR: reverse transcriptase and quantitative polymerase chain reaction
TALE: three amino-acid loop extension
UTR: untranslated region
WISH: whole mount *in situ* hybridisation
