# Supplementary material for: Retinoic acid-independent expression of Meis2 during autopod patterning in the developing bat and mouse limb
Source: EvoDevo. 2015 Mar 14;6:6. doi: 10.1186/s13227-015-0001-y (PMC4389300; doi:10.1186/s13227-015-0001-y)
Supplement: Additional file 1: Table S1. — Summary of experimental samples. The average and standard deviation of measurements for each staging group are given. Table S2. We used a BLAST analysis to find the top hit for each gene ID in the Wang et al. [34] dataset, using an Ensembl mouse transcript as the query. Table S3. Primers used to characterise Meis2 transcripts. Table S4. PCR cycling conditions for 5′ and 3′ RACE. Table S5. Summary of RT-qPCR gene targets with accession numbers for transcripts, amplicon sizes and target region, intron size and the efficiencies (E) and standard error of the mean (s.e.m). Table S6. Summary of primers used to generate WISH probes Accession numbers or references for probes are given where relevant. Table S7. Top differentially expressed genes that were over- or under-expressed in the comparisons. Gene names denoted with a dagger (†) indicate that probes were annotated by Blat analysis. Percentage similarity (%) gives the OPERON probe sequence similarity to that of the bat (Myotis lucifugus). Asterisk indicates when comparisons are significantly different (*P < 0.05, **P < 0.01). Table S8. Summary of 5′ start site of EST clones that match AK043601, relative to Homo sapiens chromosome 15. Table S9. Summary of start sites from lncMeis2 5′ RACE reactions, mapped to chromosome 15 on the H. sapiens GRCH37 assembly. Table S10. Summary of termination sites from lncMeis2 3′ RACE reactions, mapped to chromosome 2 of the M. musculus GRCm38 assembly, contig GL429805 of the M. lucifugus Myoluc2.0 assembly and chromosome 15 of the H. sapiens GRCH37 assembly. Table S11. Summary of start sites of Meis2 5′ RACE reactions mapped to contig GL429805 of the M. lucifugus Myoluc2.0 assembly, chromosome 2 of the M. musculus GRCm38 assembly and chromosome 15 of the H. sapiens GRCH37 assembly. Table S12. Genbank Accession numbers for the Meis2 overlap clones. [file 13227_2015_1_MOESM1_ESM.docx]

**Supplementary Tables**

| Table S1: Summary of experimental samples and their downstream applications. The average and standard deviation of measurements for each staging group are given. | | | | | | |
| --- | --- | --- | --- | --- | --- | --- |
| **Sample Code** | **Stage (CS)** | **Biological Repeat** | **Experiment** | **Uterus Length (mm)** | **CR Length (mm)** | **Embryo Weight (mg)** |
| MN35 | 15 | 1 | qRT-PCR | 8.2  ±0.3 | 8.2  ±0.8 | 0.11  ±0.02 |
| MN38 | 15 | 2 |  |  |  |  |
| MN46 | 15 | 3 |  |  |  |  |
| MN60 | 16 | 1 | Microarray / qRT-PCR | 8.5  ±0.5 | 9.3  ±0.3 | 0.15  ±0.02 |
| MN49 | 16 | 2 |  |  |  |  |
| MN59 | 16 | 3 |  |  |  |  |
| MN39 | 16 | 4 | Microarray |  |  |  |
| MN45 | 17 | 1 | Microarray / qRT-PCR | 8.8  ±0.6 | 10.6  ±0.5 | 0.20  ±0.04 |
| MN44 | 17 | 2 |  |  |  |  |
| MN31 | 17 | 3 |  |  |  |  |
| MN40 | 17 | 4 | Microarray |  |  |  |
| MN56 | 18 E | 1 | qRT-PCR | 9.8  ±0.8 | 12.7  ±2.1 | 0.36  ±0.09 |
| MN32 | 18 L | 2 |  |  |  |  |
| MN58 | 18 VL | 3 |  |  |  |  |

| Table S2: We used a BLAST analysis to find the top hit for each gene in the Wang et al (2014) dataset, using an Ensembl (release 75) mouse transcript as the query. The top hits that had an Eval > 1e-100, Grade > 45% and a pairwise similarity > 75% are shown. If the same sequence hit found had an alternative annotation, the annotated sequence was blasted against the NCBI nucleotide database. If the top hit did not match the query this was discarded (grey). | | | | |
| --- | --- | --- | --- | --- |
| Gene (Ensembl ID) | Gene ID | Eval | Grade (%) | Pairwise ID (%) |
| *Meis1*  (ENSMUST00000068264) | comp14476_c0_seq1 | 0 | 67.3 | 94.7 |
|  | comp13769_c0_seq1 | 0 | 66.5 | 92.8 |
| *Meis2*  (ENSMUST00000110907) | comp4463_c0_seq1 | 0 | 83.5 | 92.3 |
|  | comp4463_c0_seq2 | 0 | 83.1 | 91.6 |
| *Meis3*  (ENSMUST00000176506) | comp12264_c0_seq1 | 0 | 79.4 | 78 |
| *Rdh10*  (ENSMUST00000027053) | comp2095_c0_seq1 | 0 | 72.6 | 78.1 |
| *Aldh1a2*  (ENSMUST00000034723) | comp3518_c0_seq1 | 0 | 79.5 | 87.4 |
|  | comp3518_c0_seq2 | 0 | 79.5 | 87.4 |
| *Cyp26b1*  (ENSMUST00000077705) | comp6517_c0_seq1 | 0 | 61.3 | 85.6 |
|  | comp6517_c0_seq2 | 0 | 57.8 | 84.2 |
|  | comp2661_c0_seq1 | 0 | 48.8 | 84.4 |
| *Rarb*  (ENSMUST00000063750) | comp848_c0_seq2 | 0 | 92.3 | 84.5 |
|  | comp848_c0_seq1 | 0 | 92.3 | 84.5 |

## Table S3 Primers used to characterise *Meis2* transcripts in bat and mouse embryos

| **Primer Name** | **Primer Sequence 5’-3’** |
| --- | --- |
| 5’ RACE |  |
| GSP3_5’ RACE | ATCCCTCCCTCTCTCGCTCGTTCTCACTCGCGCT |
| NGSP3_5’ RACE | ATCGCTCTCTCGCGCTCGCTCTCTCTCGCTCTCT |
| GSP4_5’RACE | ATGCAGGCCGGATTCCCATGTGTTGCTGACC |
| NGSP4_5’ RACE | ACGGATGTGTGAGATGCTGGAAGAGCCACGC |
| GSP5_5’RACE | ATGCTGTTGTCTCCACTCTGGGAAGC |
| NGSP5_5’ RACE | ATTGAGGTTGCGTCATCGTGGTCTC |
| 3’ RACE |  |
| GSP1_3’ RACE | ACGGGCTATGGCCACCACGACTTCCGGGTTCC |
| NGSP1_3’ RACE | ACGAACCGCGCCGCCAAACTGAGGCTCTTCTA |
| GSP2_3’ RACE | ATGACGCAACCTCCACCCACTCAGCAGGCACC |
| NGSP2_3’ RACE | AGCTTCCCAGAGTGGAGACAACAGCAGTGAGCAAGGG |
| GSP7_3’ RACE | CCGCTCGTAACCTTCAGTTCGGG |
| NSGP7_3’ RACE | AGGAGTAAAGGAGGAGGAGGAAGATCA G |
| Mapping Meis2 5’ UTR |  |
| Meis2Overlap_F | GGGAGGAAGAATTCAAGAAGC |
| Meis2Overlap_R | CCAGTCCGGATAAGAAAGTGA |
| Meis2_F | GAAGAAACAGTTAGCGCAAGACA |
| Meis2_R | ACCATCCAACACAAAGCTCC |
| lncMeis2_F | CTATGGCCACCACGACTTC |
| lncMeis2_R | TGTCAGTAGGTGTTGGCAGG |

## Table S4: PCR Cycling conditions, T_m_ (melting temperature), T_a_ (annealing temperature) and T_e_ (extension temperature) for 5’ and 3’ RACE

|  | **Tm** | **Ta** | **Te** | **No cycle** |
| --- | --- | --- | --- | --- |
| 1. Primary RACE PCR |  |  |  |  |
| 1.1 | 94°C 30 sec | 72°C 3 min |  | 5 |
| 1.2 | 94°C 30 sec | 70°C 30 sec | 72°C 3 min | 5 |
| 1.3 | 94°C 30 sec | 68°C 30 sec | 72°C 3 min | 27 |
| 2. Nested RACE PCR |  |  |  |  |
| 2.1 | 94°C 30 sec | 68°C for 30 sec | 72°C 3 min | 20 |
| 3. High Fidelity PCR |  |  |  |  |
| 3.1 | 95°C 3 min |  |  |  |
| 3.2 | 98°C 20 sec | 60°C for 15 sec | 72°C 1 min | 30 |
| 3.3 |  |  | 72°C 1 min |  |

##

| Table S5: Summary of RT-qPCR gene targets giving accession numbers for transcripts, amplicon sizes and target region, intron size and the efficiencies (E) and standard error of the mean (s.e.m) for each primer set. | | | | | | | | |
| --- | --- | --- | --- | --- | --- | --- | --- | --- |
| **Gene** | **Operon Probe**  **(Accession No)** | | **Primer Sequence (5’- 3’)** | | **Target Region** | **Amplicon Size (bp)** | **Intron Size (bp)** | **E**  **±SE_mean_** |
| 5'-*Meis2* | M400017713 | F: 5’-CTATGGCCACCACGACTTC | | 5’ of Locus | | 118 | 0 | 1.00  ±0.01 |
|  |  | R: 5’-TGTCAGTAGGTGTTGGCAGG | |  |  |  |  |  |
| 3’-*Meis2* | M200002041 | F: 5’-GAAGAAACAGTTAGCGCAAGACA | | 3’ Coding | | 175 | 56 000 | 1.03  ±0.06 |
|  |  | R:5’-ACCATCCAACACAAAGCTCC | |  |  |  |  |  |
| *Tbpl1* | M300001906 | F:5’-GGCAGACAGTGATGTTGCATTGGAC | | 5’ coding | | 190 | 2 500 | 0.97  ±0.03 |
|  |  | R:5’GGTTCCTGAGGACCAAATTGTAGCTG | |  |  |  |  |  |

## Table S6: Summary of primers used to generate WISH probes for the bat (*Miniopterus natalensis*) and mouse (*Mus musculus*) giving accession numbers or references for probes where relevant.

| **Gene** | **Primer Name** | | **Sequence (5’- 3’)** | **Genbank Reference**  **(***M. natalensis***)** | **Genbank Reference (***M. musculus***)** | |
| --- | --- | --- | --- | --- | --- | --- |
| *5’Meis2* | lnc-M2_F | CTCGGCGCGGCGCGCTCC | | KM974642^*1^ | | AK043601 |
|  | lnc-M2_R | CAGTAAAAACTCCGCGAGGGGTTTCTGCGTC | |  |  |  |
| *3’Meis2* | Meis2-F | ACCCGTTGTTTCCTCTGTTAGCTCT | | KM974643^*1^ | | KM974644^*1^ |
|  | Meis2-R | GCATGAATGTCCATAACCTGTCCGC | |  |  |  |
| *Rdh10* | bRdh10-Fwd | AATGGCGAGGAAGAAATCCT | | KM974645^*1^ | | (Sandell et al., 2007) |
|  | bRdh10-Rvs | AACCGATACATGCACACGAC | |  |  |  |
| *Raldh2* | bRaldh2-Fwdi | AGGATAAGCTCGCAGACTTGG | | KM974646^*1^ | | (Mic et al., 2002) |
|  | bRaldh2-Rvsii | CTCACAAACTCCTCGTAGATGG | |  |  |  |
| *Cyp26b1* | bCyp26b1-Fwd | AGGCCATCAACGTGTACCAG | | KM974647^*1^ | | (MacLean et al., 2001) |
|  | bCyp26b1-Rvs | AGAAGGGGAGGTAATGGAAGC | |  |  |  |
| *Rarb* | bRarb-Fwd | CAGAAGTGCTTTGAAGTGGG | | KM974648^*1^ | | (Giguere et al., 1990) |
|  | bRarb-Rvs | GAATGAGAGGTGGCATTGATCC | |  |  |  |

^*1^ This study

**References**

Giguere, V., Lyn, S., Yip, P., Siu, C. H., Amin, S., 1990. Molecular-Cloning of cDNA-Encoding a 2nd Cellular Retinoic Acid-Binding Protein. Proc Natl Acad Sci U S A 87**,** 6233-6237.

MacLean, G., Abu-Abed, S., Dolle, P., Tahayato, A., Chambon, P., Petkovich, M., 2001. Cloning of a novel retinoic-acid metabolizing cytochrome P450, Cyp26B1, and comparative expression analysis with Cyp26A1 during early murine development. Mech Dev. 107**,** 195-201.

Mic, F. A., Haselbeck, R. J., Cuenca, A. E., Duester, G., 2002. Novel retinoic acid generating activities in the neural tube and heart identified by conditional rescue of Raldh2 null mutant mice. Development. 129**,** 2271-2282.

Sandell, L. L., Sanderson, B. W., Moiseyev, G., Johnson, T., Mushegian, A., Young, K., Rey, J. P., Ma, J. X., Staehling-Hampton, K., Trainor, P. A., 2007. RDH10 is essential for synthesis of embryonic retinoic acid and is required for limb, craniofacial, and organ development. Genes Dev. 21**,** 1113-24.

| Table S7: Top differentially expressed genes that were over (positive FC) or under-expressed (negative FC) in the comparisons. Gene names denoted with a dagger (^†^) indicate that probes were annotated by Blat analysis. Percentage similarity (%) gives the OPERON probe sequence similarity to that of the bat (*Myotis lucifugus*). Asterix indicates when comparisons are significantly different (* p < 0.05, ** p < 0.01). | | | | | | | |
| --- | --- | --- | --- | --- | --- | --- | --- |
| OPERON Probe | Gene Symbol [MGI Acc.] | Gene Name | Function (GO: Biological Process) | % | CS16FL / CS16HL | CS17FL / CS17HL | CS17FL / E13.5 FL |
| DE In multiple comparisons | | | | | | | |
| M400017713 | Meis2^†^ [MGI:108564] | Meis homeobox 2 | regulation of transcription, DNA-dependent (GO:0006355) | 96 | 1.9** | 4.5** | 9.9** |
| M200002041 | Hoxd11 [MGI:96203] | homeo box D11 | skeletal development (GO:0001501) | 99 | 1.8** | 2.3** | 1.7 |
| M400006624 | Gm10059^†^ [MGI:3642158] | predicted pseudogene 10059 | NA | - | 1.4 | -2.0** | -4.1** |
| M300002857 | Hapln1 [MGI:1337006] | hyaluronan and proteoglycan link protein 1 | cell adhesion (GO:0007155) | 89 | 1.2 | 1.6** | 3.1** |
| M200015556 | Cdk3-ps^†^ [MGI:1916931] | cyclin-dependent kinase 3, pseudogene | NA | - | -1.1 | 1.8** | 3.2** |
| M300006119 | 4930473A06Rik [MGI:1922152] | RIKEN cDNA 4930473A06 gene | NA | 83 | -1.1 | 1.7* | 2.7* |
| M400001515 | Taok3 [MGI:3041177] | TAO kinase 3 | protein amino acid autophosphorylation (GO:0046777) | 91 | -1.1 | 1.6* | 2.3* |
| M300005520 | Syt13 [MGI:1933945] | synaptotagmin XIII | vesicle-mediated transport (GO:0016192) | - | -1.2 | 2.0** | 3.9** |
| M400005885 | Casc1^†^ [MGI:2444480] | cancer susceptibility candidate 1 | NA | - | -1.2 | 1.8** | 5.6** |
| M400008298 | Unknown | Unknown | NA | - | -1.2 | 1.6* | 2.9** |
| M400014003 | 1700125D06Rik^†^ [MGI:1915483] | RIKEN cDNA 1700125D06 gene | NA | 83 | -1.4 | 1.8** | 4.2** |

| **OPERON Probe** | **Gene Symbol [MGI Acc.]** | **Gene Name** | **Function (GO: Biological Process)** | **%** | **CS16FL / CS16HL** | **CS17FL / CS17HL** | **CS17FL / E13.5 FL** |
| --- | --- | --- | --- | --- | --- | --- | --- |
| **DE Between 17FL and E13.5FL only** | | | | | | | |
| M300003019 | Kpna3 [MGI:1100863] | karyopherin (importin) alpha 3 | protein import into nucleus (GO:0006606) | 93 | 1 | 1.6 | 4.3** |
| M400006019 | BC049702 [MGI:3037654] | cDNA sequence BC049702 | NA | - | 1.3 | 1.3 | 3.8** |
| M200007986 | Lnp [MGI:1918115] | limb and neural patterns | embryonic digit morphogenesis (GO:0042733) | 94 | 1.1 | 1.2 | 3.3** |
| M200007994 | Kctd 1 [MGI:1918269] | potassium channel tetramerisation domain containing 1 | negative regulation of transcription (GO:0016481) | 85 | -1.1 | 1.1 | 2.9** |
| M400012306 | Mex3b [MGI:1918252] | mex3 homolog B (C. elegans) | NA | - | 1.2 | -1 | -2.5** |
| M400007165 | Hist1h2ao [MGI:2448302] | histone cluster 1, H2ao | chromosome organization and biogenesis (GO:0007001) | 90 | 1.3 | -1.4 | -2.9** |
| M400012457 | Hist1h2ao [MGI:2448302] | histone cluster 1, H2ao | chromosome organization and biogenesis (GO:0007001) | 99 | 1.1 | 1 | -2.9** |
| M400014628 | Ubr2 [MGI:1861099] | ubiquitin protein ligase E3 component n-recognin 2 | ubiquitin-dependent protein catabolic process (GO:0006511) | - | 1.3 | 1.2 | -3.0** |
| **DE Between 16FL and CS16HL only** | | | | | | | |
| M400002268 | Ajap1^†^ [MGI:2685419] | adherens junction associated protein 1 | cell adhesion (GO:0007155) | - | 1.6** | 1.2 | 2.6 |
| M400000669 | Npm1^†^ [MGI:106184] | nucleophosmin 1 | regulation of DNA damage response, signal transduction by p53 class mediator (GO:0043516) | 90 | 1.6** | -1.1 | -1.1 |
| M400013559 | Prkcd^†^ [MGI:97598] | protein kinase C, delta | positive regulation of apoptosis (GO:0043065) | - | -2.0** | 1.4 | 3 |
| M300016981 | Sbsn [MGI:2446326] | suprabasin | epidermal differentiation (Park et al, 2002) | - | -2.1** | -1.2 | 1 |

| **OPERON Probe** | **Gene Symbol**  **[MGI Acc.]** | **Gene Name** | **Function (GO: Biological Process)** | **%** | **CS16FL / CS16HL** | **CS17FL / CS17HL** | **CS17FL / E13.5 FL** |
| --- | --- | --- | --- | --- | --- | --- | --- |
| **DE Between 17FL and CS17HL only** | | | | | | | |
| M200000546 | Matn1 [MGI:106591] | matrilin 1, cartilage matrix protein 1 | extracellular matrix organization and biogenesis (GO:0030198) | 90 | -1.3 | 1.8** | 1.5 |
| M400018107 | 4930428O21Rik^†^ [MGI:1923034 ] | RIKEN cDNA 4930428O21 gene | NA | - | -1.2 | 1.8** | -1.9 |
| M300012403 | Zfp800 [MGI:1889334] | zinc finger protein 800 | regulation of transcription (GO:0045449) | 91 | -1.1 | 1.8* | 1.6 |
| M200003355 | Pgcp [MGI:1889205] | plasma glutamate carboxypeptidase | proteolysis (GO:0006508) | 92 | 1.1 | 1.7** | 1.6 |
| M300009692 | Secisbp2l [MGI:1917604] | SECIS binding protein 2-like | NA | 87 | 1.1 | 1.7** | 2.2 |
| M400013993 | 1700047M11Rik^†^ [MGI:1914580] | RIKEN cDNA 1700047M11 gene | NA | - | -1.3 | 1.7** | 2.2 |
| M200010758 | Tmc1 [MGI:2151016] | transmembrane channel-like gene family 1 | sensory perception of sound (GO:0007605) | - | -1.1 | 1.7* | 1.4 |
| M400018137 | Unknown [AK038214] | Mus musculus 16 days neonate thymus cDNA, RIKEN full-length enriched library | NA | - | 1.2 | 1.7* | -1.7 |
| M400010738 | Hrk [MGI:1201608] | harakiri, BCL2 interacting protein (contains only BH3 domain) | regulation of apoptosis (GO:0042981) | - | -1.3 | 1.6* | 1.6 |
| M400011422 | Dab2 [MGI:109175] | disabled homolog 2 (Drosophila) | cellular morphogenesis during differentiation (GO:0000904) | - | 1.1 | 1.6* | 1.3 |
| M400011967 | Olfr1303 [MGI:3031137] | olfactory receptor 1303 | G-protein coupled receptor protein signaling pathway (GO:0007186) | - | 1.2 | 1.5* | 1.4 |
| M400007409 | Rbm10^†^ [MGI:2384310 ] | RNA binding motif protein 10 | NA | - | 1.5 | -1.8** | 1 |

| Table S8: Summary of 5’ start site of human and mouse EST clones that are highly similar to AK043601, on *H. sapiens* chromosome 15. | | | | |
| --- | --- | --- | --- | --- |
| EST Genbank Reference | EST library code | | Tissue Type | Start site relative to *H. sapiens* GRCh37: Chr15 |
| Human EST library clones | | | | |
| DA249768 | BRAWH3 | | normal astrocytes | 37392750 |
| DA724781 | NT2RI3 | | NT2 neuronal cells | 37392750 |
| DB291655 | UTERU3 | | fetal brain | 37392750 |
| DA030451 | ASTRO2 | | normal astrocytes | 37392750 |
| DA690076 | NT2NE2 | | NT2 neuronal cells | 37392750 |
| DA772261 | OCBBF2 | | NT2 neuronal cells | 37392750 |
| DB292525 | UTERU3 | | fetal brain | 37392750 |
| DA757216 | NT2RP8 | | NT2 neuronal cells | 37392750 |
| DA712206 | NT2RI2 | | NT2 neuronal cells | 37392750 |
| DA757110 | NT2RP8 | | NT2 neuronal cells | 37392750 |
| DB285787 | UTERU3 | | fetal brain | 37392749 |
| DA754391 | NT2RP8 | | NT2 neuronal cells | 37392749 |
| DA725085 | NT2RI3 | | NT2 neuronal cells | 37392749 |
| DA770123 | OCBBF2 | | NT2 neuronal cells | 37392749 |
| DA517866 | FEBRA2 | | normal astrocytes | 37392749 |
| DA773586 | OCBBF2 | | NT2 neuronal cells | 37392749 |
| DA776234 | OCBBF2 | | NT2 neuronal cells | 37392749 |
| DA501601 | FCBBF3 | | normal astrocytes | 37392749 |
| DA758456 | NT2RP8 | | NT2 neuronal cells | 37392749 |
| DA493748 | FCBBF3 | | normal astrocytes | 37392749 |
| DA256621 | BRCAN2 | | normal astrocytes | 37392749 |
| DA741733 | NT2RP7 | | NT2 neuronal cells | 37392749 |
| DA801790 | OCBBF3 | | fetal brain | 37392749 |
| DA483678 | FCBBF2 | | normal astrocytes | 37392749 |
| DA776561 | OCBBF2 | | NT2 neuronal cells | 37392749 |
| DB289759 | UTERU3 | | fetal brain | 37392744 |
| DA739568 | NT2RP7 | | NT2 neuronal cells | 37392741 |
| DC371533 | NT2RP7 | | fetal brain | 37392740 |
| DA701566 | NT2RI2 | | NT2 neuronal cells | 37392740 |
| DA723217 | NT2RI3 | | NT2 neuronal cells | 37392740 |
| DA490618 | FCBBF3 | | normal astrocytes | 37392740 |
| DA759471 | NT2RP8 | | NT2 neuronal cells | 37392738 |
| DA779276 | OCBBF2 | | fetal brain | 37392736 |
| DA784094 | OCBBF2 | | fetal brain | 37392709 |
| Mouse EST library clones | | | | |
| CJ131445 | | RIKEN, | 10 day neonate cortex | 37392737 |
| BB641212 | | RIKEN, | 10 day neonate cortex | 37392731 |
| BB263500 | | RIKEN | 10 day neonate cortex | 37392731 |
| BE291952 | | NCI_CGAP_Mam6 | mammary gland_infiltrating ductal carcinoma | 37392689 |
| BX528066 | | NCI_CGAP_Mam6 | mammary gland_infiltrating ductal carcinoma | 37392689 |
| BB854874 | | RIKEN | B16 F10Y cells | 37392631 |
| BY228744 | | RIKEN | bone marrow mast cells | 37392602 |

## Table S9: Summary of start sites of clones from bat and mouse *lncMeis2* 5’ RACE reactions, mapped to chromosome 15 on the *H. sapiens* GRCH37 assembly.

| EST Genbank Reference | 5' RACE clone name | | Start site relative to *H. sapiens* GRCh37:Chr15: (- strand) |
| --- | --- | --- | --- |
| *M. natalensis forelimb* | | |  |
| KM609410 | BF5ngsp3_A_e | | 37392759 |
| KM609411 | BF5ngsp3_A_a | | 37392749 |
| KM609412 | BF5ngsp3_A_d | | 37392749 |
| KM609413 | BF5ngsp3_A_b | | 37392748 |
| KM609414 | BF5ngsp3_A_j | | 37392748 |
| KM609415 | BF5ngsp3_A_h | | 37392747 |
| KM609416 | BF5ngsp3_A_i | | 37392741 |
| KM609417 | BF5ngsp3_A_g | | 37392735 |
| *M. natalensis head* | | |  |
| KM974624 | BH5ngsp3_A_f | | 37392749 |
| KM974626 | BH5ngsp3_A_h | | 37392748 |
| KM974625 | BH5ngsp3_A_g | | 37392740 |
| KM974627 | BH5ngsp3_A_i | | 37392734 |
| KM974620 | BH5ngsp3_A_a | | 37392733 |
| KM974621 | BH5ngsp3_A_b | | 37392733 |
| KM974622 | BH5ngsp3_A_d | | 37392731 |
| KM974628 | BHngsp3_A_j | | 37392726 |
| KM974623 | BHngsp3_A_e | | 37392724 |
| *M.musculus forelimb* | | |  |
| KM974634 | MF5ngsp3_A_m | | 37392747 |
| KM974629 | MF5ngsp3_A_b | | 37392747 |
| KM974631 | MF5ngsp3_A_d | | 37392743 |
| KM974633 | MF5ngsp3_A_l | | 37392734 |
| KM974632 | MF5ngsp3_A_k | | 37392723 |
| *M. musculus head* | | | |
| KM974639 | MHngsp3_A-g | 37392753 | |
| KM974635 | MHngsp3_A-a | 37392748 | |
| KM974637 | MH ngsp3_A-e | 37392743 | |
| KM974640 | MH ngsp3_A-h | 37392743 | |
| KM974636 | MH ngsp3_A-d | 37392740 | |
| KM974641 | MHngsp3_A-j | 37392735 | |
| KM974630 | MHngsp3_A-c | 37392734 | |
| KM974638 | MH ngsp3_A-f | 37392729 | |

## Table S10: Summary of termination sites of clones from bat and mouse *lncMeis2* 3’ RACE reactions, mapped to contig GL429805 of the *M. lucifugus* Myoluc2.0 assembly, chromosome 2 of the *M. musculus* GRCm38 assembly, and chromosome 15 of the *H. sapiens* GRCH37 assembly.

| Genbank Accession Number | Clone Name | *M. lucifugus* genome Myoluc2.0 :GL429805 termination site | *M. musculus* genome GRCm38:chr2  termination site | *H. sapiens* GRCh37:Chr 15 termination site |
| --- | --- | --- | --- | --- |
|  | GSP1/NGSP1 primers |  |  |  |
|  | *M. natalensis forelimb* |  |  |  |
| KP101222 | BF3ngsp1_A_a | 6317800 | 116065358 | 37392233 |
| KP101223 | BF3ngsp1_A_b | 6317799 | 116065357 | 37392232 |
| KP101224 | BF3ngsp1_A_c | 6317800 | 116065358 | 37392233 |
| KP101225 | BF3ngsp1_A_d | 6317796 | 116065354 | 37392229 |
| KP101226 | BF3ngsp1_A_e | 6317796 | 116065354 | 37392233 |
| KP101227 | BF3ngsp1_A_f | 6317800 | 116065358 | 37392233 |
| KP101228 | BF3ngsp1_A_g | 6317796 | 116065354 | 37392229 |
| KP101229 | BF3ngsp1_A_h | 6317796 | 116065354 | 37392229 |
| KP101230 | BF3ngsp1_A_i | 6317800 | 116065358 | 37392233 |
| KP101231 | BF3ngsp1_A_j | 6317796 | 116065354 | 37392229 |
|  | *M. natalensis head* |  |  |  |
| KP101232 | BH3ngsp1_A_b | 6317796 | 116065354 | 37392229 |
| KP101233 | BH3ngsp1_A_c | 6317796 | 116065354 | 37392229 |
| KP101234 | BH3ngsp1_A_d | 6317796 | 116065354 | 37392229 |
| KP101235 | BH3ngsp1_A_e | 6317796 | 116065354 | 37392229 |
| KP101236 | BH3ngsp1_A_f | 6317799 | 116065357 | 37392232 |
| KP101237 | BH3ngsp1_A_g | 6317799 | 116065357 | 37392232 |
| KP101238 | BH3ngsp1_A_i | 6317796 | 116065354 | 37392229 |
| KP101239 | BH3ngsp1_A_j | 6317801 | 116065359 | 37392234 |
|  | GSP1/NGSP1 primers |  |  |  |
|  | *M. musculus forelimb* |  |  |  |
| KM974602 | MF3ngsp1_A_b | 6317799 | 116065357 | 37392232 |
| KM974603 | MF3ngsp1_A_e | 6317796 | 116065354 | 37392229 |
| KM974604 | MF3ngsp1_A_f | 6317796 | 116065354 | 37392229 |
| KM974605 | MF3ngsp1_A_g | 6317799 | 116065357 | 37392232 |
| KM974606 | MF3ngsp1_A_h | 6317799 | 116065357 | 37392232 |
| KM974607 | MF3ngsp1_A_k | 6317375 | 116064948 | 37391809 |
| KM974610 | MF3ngsp1_B_c | 6317800 | 116065358 | 37392233 |
| KM974611 | MF3ngsp1_B_d | 6317800 | 116065358 | 37392233 |
| KM974614 | MF3ngsp1_B_f | 6317800 | 116065358 | 37392233 |
| KM974616 | MF3ngsp1_B_g | 6317843 | 116065401 | 37392277 |
|  | *M. musculus head* |  |  |  |
| KM974608 | MH3ngsp1_B_a | 6317801 | 116065391 | 37392236 |
| KM974609 | MH3ngsp1_B_b | 6317801 | 116065391 | 37392236 |
| KM974613 | MH3ngsp1_B_d | 6317799 | 116065357 | 37392232 |
| KM974612 | MH3ngsp1_B_e | 6317821 | 116065379 | 37392254 |
| KM974615 | MH3ngsp1_B_f | 6317799 | 116065357 | 37392232 |
| KM974617 | MH3ngsp1_B_g | 6317800 | 116065358 | 37392233 |
| KM974618 | MH3ngsp1_B_i | 6317799 | 116065357 | 37392232 |

## Table S11: Summary of start sites of *Meis2* 5’ RACE transcripts mapped to contig GL429805 of the *M. lucifugus* Myoluc2.0 assembly, chromosome 2 of the *M. musculus* GRCm38 assembly, and chromosome 15 of the *H. sapiens* GRCH37 assembly.

| Genbank Accession Number | Primers used and Clone Name | *M. lucifugus* genome Myoluc2.0 :GL429805 start site | *M. musculus* genome GRCm38:chr2  start site | *Hsapiens* GRCh37:Chr 15 start sites |
| --- | --- | --- | --- | --- |
|  | GSP4/NGSP4 primers |  |  |  |
|  | *M. natalensis forelimb* |  |  |  |
| KM974596 | BF5ngsp4A_j | 6317494 | 116065057 | 37391923 |
| KM974593 | BF5ngsp4A_b | 6317466 | 116065032 | 37391895 |
| KM974598 | BF5ngsp4B_a | 6317364 | 116064938 | 37391799 |
| KM974600 | BF5ngsp4B_d | 6317363 | 116064937 | 37391798 |
| KM974595 | BF5ngsp4A_h | 6317326 | 116064905 | 37391764 |
| KM974599 | BF5ngsp4B_c | 6317343 | 116064922 | 37391781 |
| KM974601 | BF5ngsp4B_e | 6317313 | 116064896 | 37391750 |
| KM974594 | BF5ngsp4A_e | 6317241 | 116064824 | 37391678 |
|  | *M. natalensis head* |  |  |  |
| KP101220 | BH5ngsp4A_d | 6317480 | 116065046 | 37391909 |
| KM974597 | BH5ngsp4A_h | 6317466 | 116065032 | 37391895 |

## Table S12: Genbank Accession numbers for the *Meis2* overlap clones

| Genbank Accession Number | Tissue Type | Primers used and Clone Name | Clone name |
| --- | --- | --- | --- |
| KM974592 | *M. natalensis head* | Meis Overlap F&R | *M. natalensis* *Meis2*_overlap |
| KP101221 | *Mus musculus head* | Meis Overlap F&R | *M musculus* *Meis2* overlap |
