# Supplementary material for: Retinoic acid-independent expression of Meis2 during autopod patterning in the developing bat and mouse limb
Source: EvoDevo. 2015 Mar 14;6:6. doi: 10.1186/s13227-015-0001-y (PMC4389300; doi:10.1186/s13227-015-0001-y)
Supplement: Additional file 2: Figure S1. — Summary of the positions of each RACE primer and PCR product relative to the bat Meis2 locus. The red blocks indicate the different bat EST clone boundaries corresponding to the different 5′ and 3′ RACE and PCR reactions. The green arrows represent the 3′ RACE primers and the blue arrows represent the 5′ RACE primers. The position of polyA tracts on the mRNA and genome template are indicated by black arrows. Primers used in a standard PCR reaction to amplify cDNA synthesised with random hexamers, over these polyA tracts, are shown with yellow arrows. The presence of a polyadenylation-recognition sequence (AAAUAAA), 16 nucleotides from the polyA tail, is shown. The polyA tracts are present in the genome Meis2 contig and are not preceded by a polyadenylation-recognition sequence. Additional file descriptions text (including details of how to view the file, if it is in a non-standard format). [file 13227_2015_1_MOESM2_ESM.pptx]

## Slide 1
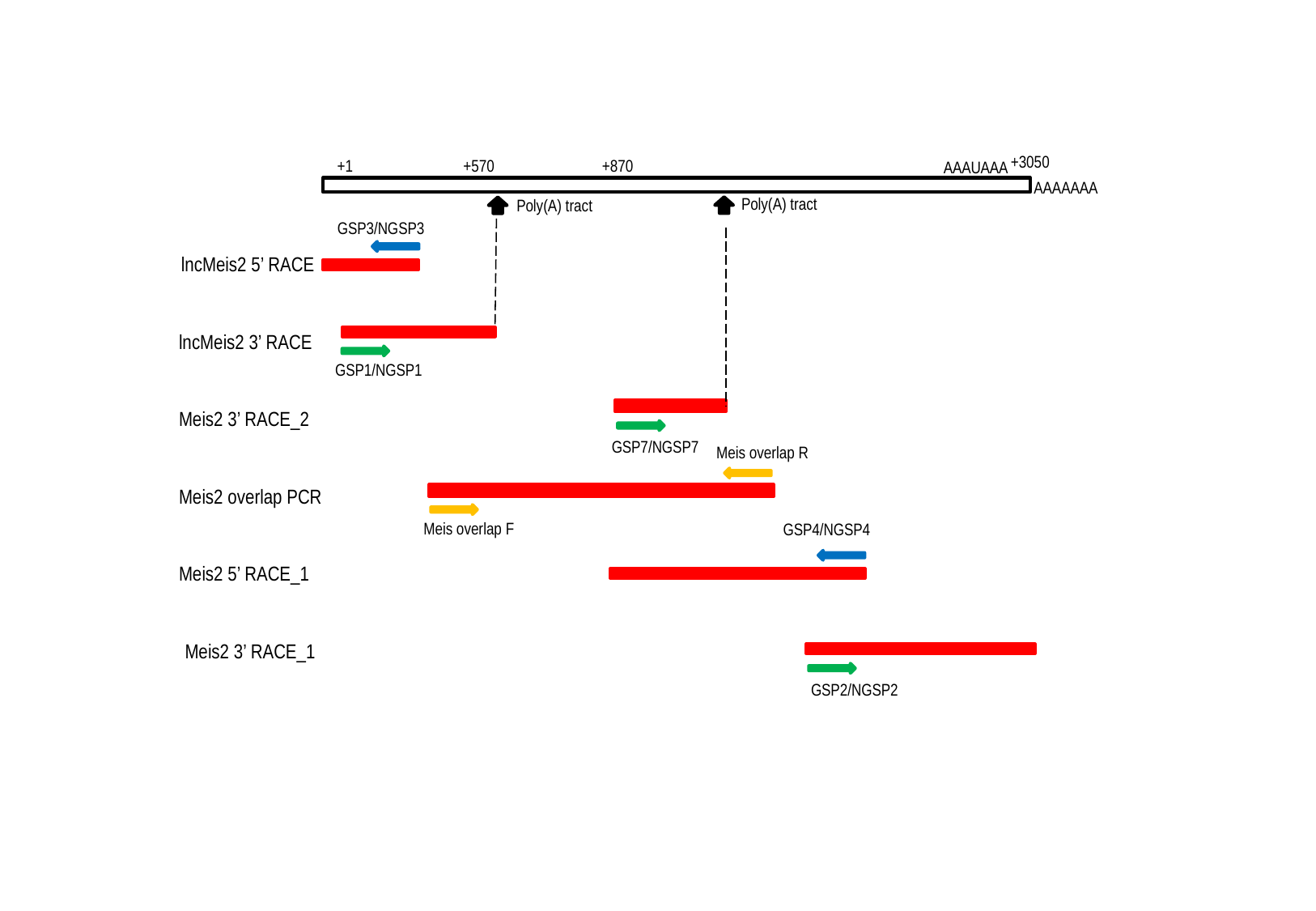

+3050
+870
+1
+570
AAAUAAA
AAAAAAA
Poly(A) tract
Poly(A) tract
GSP3/NGSP3
lncMeis2 5’ RACE
lncMeis2 3’ RACE
GSP1/NGSP1
Meis2 3’ RACE_2
GSP7/NGSP7
Meis overlap R
Meis2 overlap PCR
Meis overlap F
GSP4/NGSP4
Meis2 5’ RACE_1
Meis2 3’ RACE_1
GSP2/NGSP2
