# Supplementary material for: Retinoic acid-independent expression of Meis2 during autopod patterning in the developing bat and mouse limb
Source: EvoDevo. 2015 Mar 14;6:6. doi: 10.1186/s13227-015-0001-y (PMC4389300; doi:10.1186/s13227-015-0001-y)
Supplement: Additional file 3: — Supplementary Methods. Additional information on experimental conditions for 5′ and 3′ RACE reactions to map Meis2 transcripts and high fidelity PCR. [file 13227_2015_1_MOESM3_ESM.docx]

**Supplementary Material**

# Supplementary Methods

## 5’ and 3’ RACE

The first round of gene specific RACE reactions was carried out using Advantage™ 2 PCR kit (Clontech, USA) in a reaction mix containing 2.5µL of RACE ready cDNA, 5µL of universal RACE primer mix (10 times concentration), 1µL of gene specific primers (GSP) (10µM), 5µL of 10X Advantage 2 PCR buffer, 1µL of dNTPs (10µM), 1µL of 50X Advantage 2 Polymerase Mix in a final reaction volume of 50µL. 5’ and 3’ RACE products were amplified using PCR cycling conditions summarized in Table S5. The products of the first round RACE PCRs were diluted 1:50 in Tricine-EDTA buffer (ClonTech, USA) and used as the templates for a second round of nested RACE PCRs. The nested RACE reaction tube was setup as stated for the first round RACE reaction tube except that 2.5µL of nested GSP (NGSP) was used instead of GSP. The thermal cycling conditions for the nested RACE PCR reactions are summarized in Table S4. The nested RACE PCR products were resolved on a 2% (w/v) agarose gels, and prominent single bands were excised from the gel, and purified using the Wizards® SV Gel and PCR clean-up kit (Promega, USA). The purified PCR products were cloned into the pGEM®-T Easy vector system (Promega, USA). Recombinant plasmids were sequenced by Sanger dideoxy-sequencing at University of Washington High-throughput Genomic Unit, USA (UWHTGU) or the Central Analytic Facility at Stellenbosch University.

## High Fidelity PCR

The Ambion DNase I treatment kit (Ambion, USA) was used to digest any genomic DNA contaminating the RNA samples; 2 µg of total RNA was digested in the presence of 1µl of 10x DNase I buffer and 1µl of rDNase I (enzyme) in a 10 μl reaction, at 37°C 20-30 minutes at 37°C. After the incubation, 2 µl of re-suspended DNAse I Inactivation Reagent was added and the reaction was then incubated at room temperature for 2 minutes with occasional mixing. The reaction tubes were then centrifuged at 10,000x g for 1.5 minutes and the supernatant containing treated RNA was collected. This DNase I treated RNA was used to synthesize cDNA using SuperScript III reverse transcriptase (RT) (Invitrogen, USA), in a reaction mix containing 1µg of the DNase-treated RNA in 5µl of solution, 1µl of 10mM dNTPs and 1 µl of 50mM random hexamers was added and the volume was topped up with DEPC treated water to 13 µl. The reaction tubes were then incubated for 10min at 25°C and then 65°C for 5 minutes; to anneal the primers to the mRNA transcripts. The reaction tubes were placed on ice and 1µl RNAseOUT ® (Invitrogen, USA) was added as well as 1µl of 1M dithiothreitol and 4µl of 5X FS buffer and 1µl SuperScript III RT. The reaction tubes were mixed on a vortex for a few seconds and incubated for 10 minutes at 25°C, 50°C for 60 min and 85°C for 5 minutes. A “no RT” reaction was used as a control to check that there was no genomic DNA contamination (- RT reaction). The cDNA synthesis and - RT reactions were used as templates with the PCR primer sets listed in Table S4 in a reaction mix containing 10µl of 5X KAPA HiFi GC buffer, 1.5µl of 10mM KAPA dNTP mix 1.5µl for each 10µM primer, 2µl of cDNA, 1µl of KAPA HiFi HotStart DNA Polymerase. The PCR cycling conditions are summarized in Table S4. PCR products were purified from 2% w/v agarose gel and cloned into the pGEM-T Easy® vectors as previously described.
