# Supplementary material for: Retinoic acid-independent expression of Meis2 during autopod patterning in the developing bat and mouse limb
Source: EvoDevo. 2015 Mar 14;6:6. doi: 10.1186/s13227-015-0001-y (PMC4389300; doi:10.1186/s13227-015-0001-y)
Supplement: Additional file 4: — Supplementary Results. Analysis showing that the microarray probe M400017713 corresponds to the 5′ UTR of Meis2 transcripts and statistical analysis of relative qPCR data for 5′-Meis2 and 3′-Meis2. [file 13227_2015_1_MOESM4_ESM.docx]

**Supplementary Material**

# Supplementary Results

# **Analysis of Meis2 transcripts: The microarray probe M400017713 corresponds to the 5’ UTR of the Meis2 mRNA transcript**.

A blastn analysis identified 31 human and 7 mouse EST clones corresponding to *lncMeis2*, which were derived mainly from embryonic head tissue or neural cell lines, (Table S8). The 5’ ends of the majority of the human ESTs (97%) mapped to GRCh37 chr15:37392750-37392730, whilst the mouse lncMeis2 EST clones mapped over a larger area between human GRCh37 chr15: 37392737-37392602 (Fig. S2). A blastn analysis of *lncMeis2* against the nr database, found matches overlapping with five human *Meis2* variants (*Meis2a-e*), the start sites of which all of which mapped to GRCh37:chr15: 37392341, 408bp away from the putative human lncMeis2 TSS, with substantial overlap with the 3’ end of the putative *lncMeis2* transcripts. Mouse *Meis2* variants in the nr database were shorter with no overlap between the mouse putative *lncMeis2* and *Meis2* transcripts.

Here we mapped the boundaries of the putative bat and mouse *lncMeis2* transcripts to confirm whether *lncMeis2* was indeed a polyadenylated, long non-coding RNA, with an independent TSS from the coding *Meis2* transcript. The 5’ and 3’ RACE reactions successfully amplified products corresponding to the putative *lncMeis2* and *Meis2* cDNAs from forelimb and head tissues taken from bat and mouse embryos (summarized in Fig S1).

The relative start sites of the majority of bat and mouse *lncMeis2* 5’ RACE clones (summarized in Table S9) overlapped the human *lncMeis2* EST clone start sites (GRCh37:chr15:37392750-37392730) Fig S2.

All the 3’ RACE clones for bat *lncMeis2* from both head and limb, terminated at the same position, which aligned exactly with adenine rich motifs on the mouse (GRCm38:chr2: 116065354), human (GRCh37:15:37392229) and *Myotis lucifugus* (Myoluc2.0:GL429805: 6317796) genomes (Table S10, Fig S1). The putative bat *lncMeis2* transcripts lacked a polyadenylation recognition sequence (AAAUAAA), which is commonly found 10-30 nucleotides before the end of polyadenylated mRNA transcripts. We completed a second 3’ RACE reaction using the GSP7/NGSP7 primers (Fig. S1) with bat head and FL cDNA. These 3’ RACE reactions also terminated at adenine rich motifs on the mouse (GRCm38:chr2:116064905), human (GRCh37:15: 37391764) and *M. lucifigus* (Myoluc2.0:GL429805: 6317355) genomes.

With one exception, the mouse *lncMeis2* 3’ RACE GSP1/NGSP1 cDNA clones terminated at the same adenine-rich sites site as the bat 3’ RACE cDNA clones (Table S10). The exception, clone MF3ngsp1_A_k, ran through the first adenine-rich domain, and terminated 425bp later, at the next adenine-rich domain (mouse (GRCm38:chr2:116064948), human (GRCh37:15: 37391809) and *M. lucifigus* (Myoluc2.0:GL429805: 6317375) genomes. This data was supported by several mouse EST clones (BY740861, BY228744, BX528066, BE291952, BB854874), which also extended through the first adenine-rich domain. Furthermore, the majority the human ESTs clones extended past the first adenine-rich sequence on the *Meis2* 5’ UTR. It is thus likely that the 3’ RACE results presented here, and the RIKEN mouse clone AK043601 were a consequence of oligo(dT) mispriming to internal adenine-rich regions of *Meis2* mRNA transcripts during cDNA synthesis (Nam et al., 2002)

The longest 5’ RACE sequences derived from embryonic bat limb and head cDNA, starting from the *Meis2* coding region (Fig S1) did not overlap with *lncMeis2* (Table S11). However, we were able to amplify and sequence the gap between the adenine-rich region, and the start of the *Meis2* 5’ RACE cDNA clones, using high fidelity PCR on mouse and bat head cDNA indicating that there is transcript coverage over this region(Fig S1, Table S12).

## Relative qPCR Statistical Analysis

RT-qPCR reactions using primer sets that amplified either the 5’ *Meis2* (*5’-Meis2*) or the 3’-*Meis2* (*3’-Meis2*) region revealed that *Meis2* transcripts were more abundant in the bat CS17 FL when compared to both the HL and the mouse E13.5 FL. This was significant for the *5’-Meis2* primer pair (Kruskal-Wallis H test: *H* = 7.2, d.f. = 2, *p* = 0.027) with pairwise comparison revealing that the CS17 FL was significantly higher the E13.5 FL (*p* = 0.022) but not the CS17 HL. For both experiments, the transcript abundance of the FL samples were over 2-fold higher than that HL from CS16 onwards, with abundance peaking in the CS7 FL. However the differences between the FL and the HL were only significant for the *3’ Meis2* experiment (Related Samples Sign test: *Statistic* = -2.021, N = 12, *p* = 0.043). When examining the FL and HL expression over the sequential stages of development, both limb type and developmental stage had an effect on *5’-Meis2* abundanc*e* (Two-way ANOVA: *F*(3,16) = 59.2, *p* < 0.001, partial η^2^ = 0.92). This was statistically significant between the limb types of the older developmental stages: CS16 (*F*(1,16) = 22.5, *p* < 0.001, partial η^2^ = 0.59), CS17 (*F*(1,16) = 252.7, *p* < 0.001, partial η^2^ = 0.94), and CS18 (*F*(1,16) = 140.2, *p* < 0.001, partial η^2^ = 0.90) and amongst the developmental stages of the FL (*F*(3,16) = 155.3, *p* < 0.001, partial η^2^ = 0.97). We could not determine the interaction effects of the *3’-Meis2* experiment as the data contained an outlier (developmental stage CS15), and did not satisfy the assumption of homogeneity of variances, both when this stage was excluded and when the data was transformed (Square Root and Log_10_). However, there was a strong positive correlation between the *5’-Meis2* expression data (Log_10_ transformed) and that of *3’-Meis2* (Pearson Correlation: *r*(22) = 0.95, *p* < 0.001).

# References

Nam, D. K., Lee, S., Zhou, G., Cao, X., Wang, C., Clark, T., Chen, J., Rowley, J. D., Wang, S. M., 2002. Oligo(dT) primer generates a high frequency of truncated cDNAs through internal poly(A) priming during reverse transcription. Proc Natl Acad Sci U S A. 99**,** 6152-6.
