# Supplementary material for: Retinoic acid-independent expression of Meis2 during autopod patterning in the developing bat and mouse limb
Source: EvoDevo. 2015 Mar 14;6:6. doi: 10.1186/s13227-015-0001-y (PMC4389300; doi:10.1186/s13227-015-0001-y)
Supplement: Additional file 5: Figure S2. — Histogram and screen-shot. Histogram (A) summarises the frequency of the 5′ boundaries of mouse and human ESTs that match lncMeis2, relative to 5′ RACE results from mouse and bat limb and head cDNA mapped to the strand of human chromosome chr15:37392600-37392800. A screen-shot (B) summarises the distribution of human and mouse Fantom5 CAGE tags that map to the equivalent region on the Zenbu browser, confirming that this region corresponds to the major TSS for Meis2. [file 13227_2015_1_MOESM5_ESM.pptx]

## Slide 1
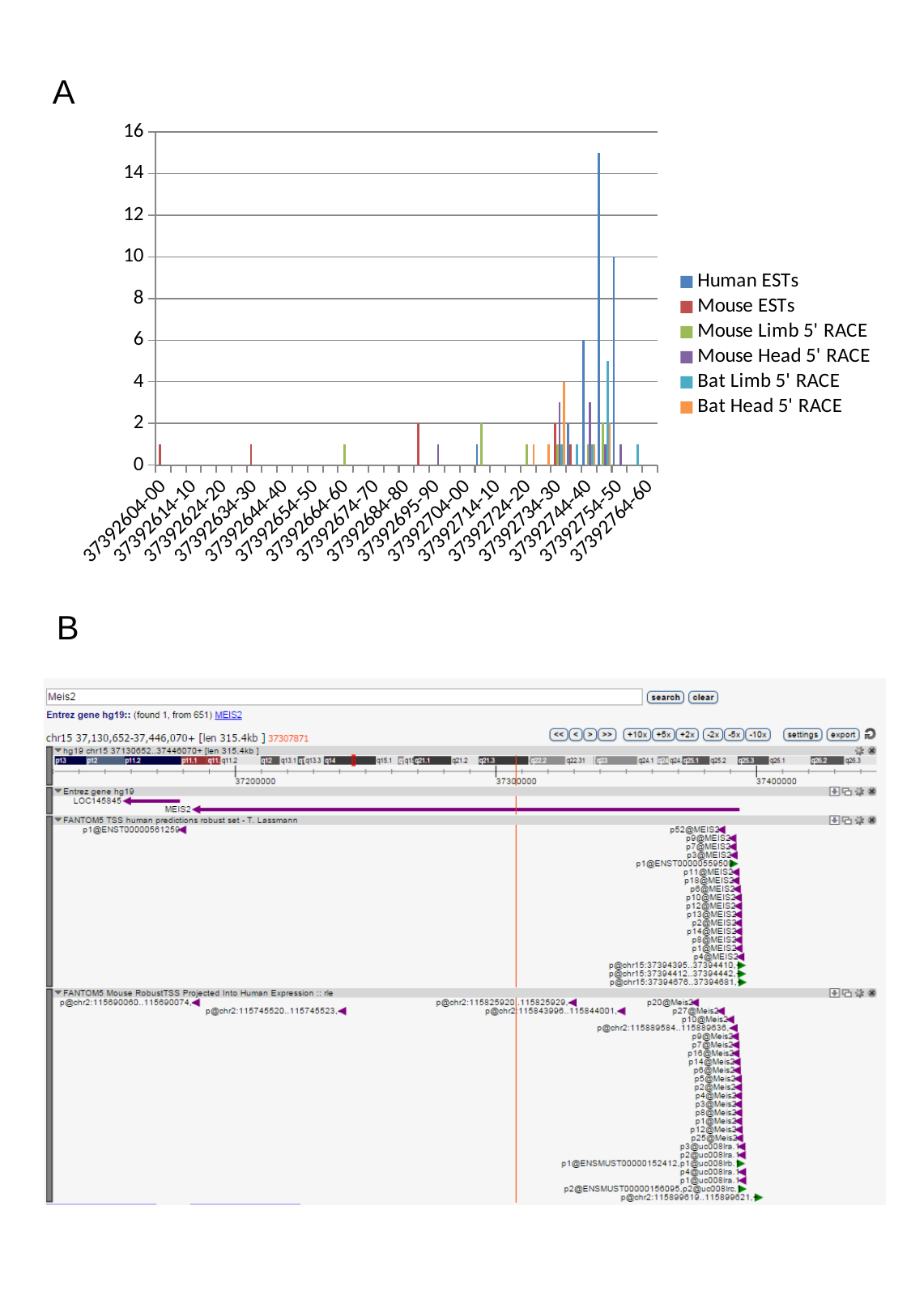

A
### Chart
| Category | Human ESTs | Mouse ESTs | Mouse Limb 5' RACE | Mouse Head 5' RACE | Bat Limb 5' RACE | Bat Head 5' RACE |
|---|---|---|---|---|---|---|
| 37392604-00 | None | 1.0 | None | None | None | None |
| 37392609-05 | None | None | None | None | None | None |
| 37392614-10 | None | None | None | None | None | None |
| 37392619-15 | None | None | None | None | None | None |
| 37392624-20 | None | None | None | None | None | None |
| 37392629-25 | None | None | None | None | None | None |
| 37392634-30 | None | 1.0 | None | None | None | None |
| 37392639-35 | None | None | None | None | None | None |
| 37392644-40 | None | None | None | None | None | None |
| 37392649-45 | None | None | None | None | None | None |
| 37392654-50 | None | None | None | None | None | None |
| 37392659-55 | None | None | None | None | None | None |
| 37392664-60 | None | None | 1.0 | None | None | None |
| 37392669-65 | None | None | None | None | None | None |
| 37392674-70 | None | None | None | None | None | None |
| 37392679-75 | None | None | None | None | None | None |
| 37392684-80 | None | None | None | None | None | None |
| 37392689-85 | None | 2.0 | None | None | None | None |
| 37392695-90 | None | None | None | 1.0 | None | None |
| 37392699-95 | None | None | None | None | None | None |
| 37392704-00 | None | None | None | None | None | None |
| 37392709-05 | 1.0 | None | 2.0 | None | None | None |
| 37392714-10 | None | None | None | None | None | None |
| 37392719-15 | None | None | None | None | None | None |
| 37392724-20 | None | None | 1.0 | None | None | 1.0 |
| 37392729-25 | None | None | None | 0.0 | None | 1.0 |
| 37392734-30 | None | 2.0 | 1.0 | 3.0 | 1.0 | 4.0 |
| 37392739-35 | 2.0 | 1.0 | None | 0.0 | 1.0 | None |
| 37392744-40 | 6.0 | None | 1.0 | 3.0 | 1.0 | 1.0 |
| 37392749-45 | 15.0 | None | 2.0 | 1.0 | 5.0 | 2.0 |
| 37392754-50 | 10.0 | None | None | 1.0 | None | None |
| 37392759-55 | None | None | None | None | 1.0 | None |
| 37392764-60 | None | None | None | None | None | None |B
