# Supplementary figures and images for: Retinoic acid-independent expression of Meis2 during autopod patterning in the developing bat and mouse limb
Source: EvoDevo. 2015 Mar 14;6:6. doi: 10.1186/s13227-015-0001-y (PMC4389300; doi:10.1186/s13227-015-0001-y)

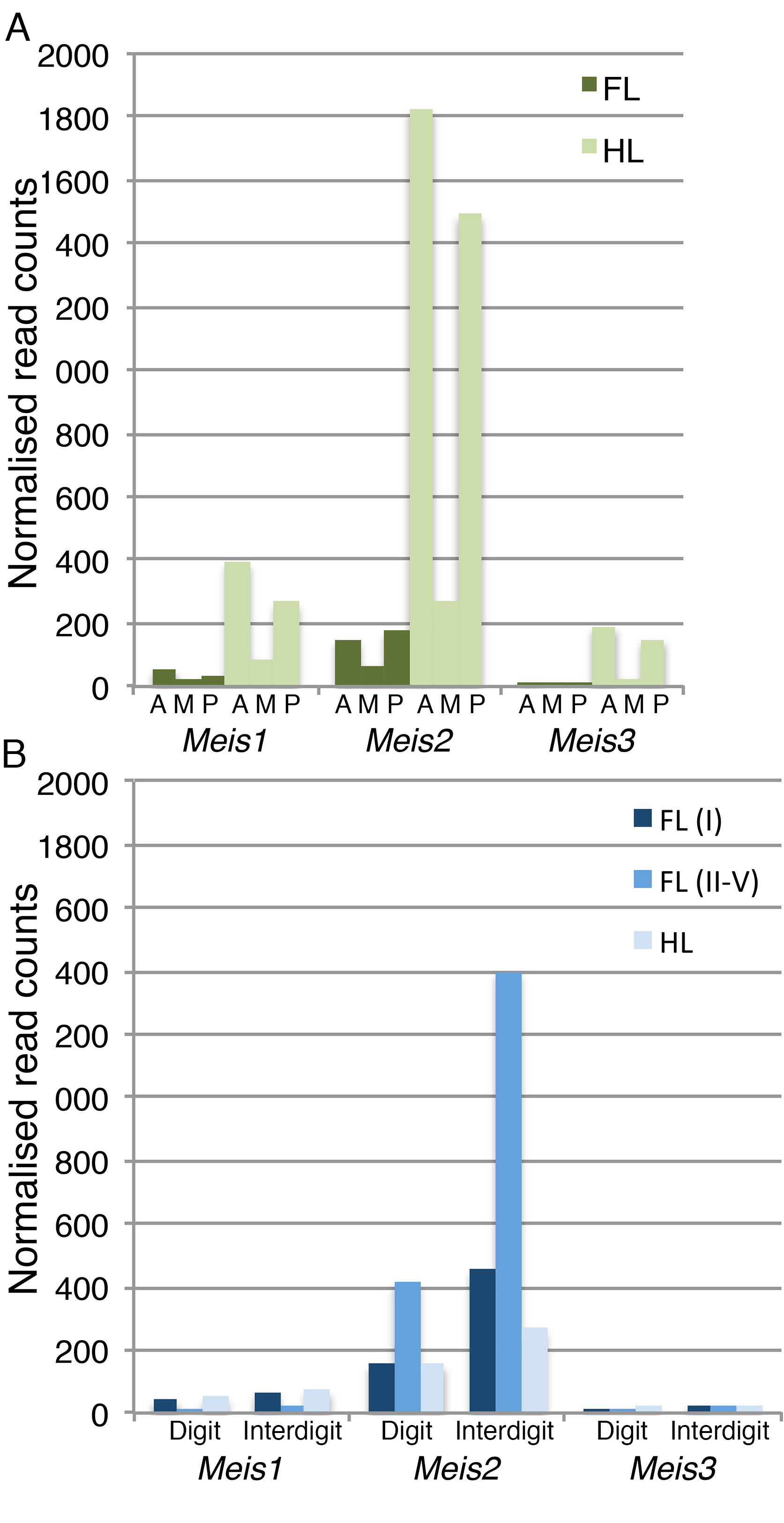

Supplement: Additional file 6: Figure S3. — RNA-seq reads for Meis2. RNA-seq reads from (A) CS14 forelimb anterior, medial and posterior (A, M and P, respectively) and their corresponding hindlimb regions show that the expressions of the Meis genes are highest in the hindlimb corresponding to the anterior and posterior limb bud region, with Meis2 being the most predominantly expressed transcript. The Meis2 transcript was also the most abundant Meis transcript in the (B) pooled CS15-CS17 limb samples that compared expression among digit and interdigit regions of the forelimb (FL) and the hindlimb (HL). The FL tissues were separated into the anterior portion (I) containing either digit I or the adjacent interdigit and the posterior portion (II to V) containing digits II to V or the corresponding interdigits. The highest expression was found in the forelimb posterior interdigits (FL (II to V)), with lowered expression occurring in the digits of FL (I), FL (II to V), interdigits FL (I) and the digits and interdigits of the hindlimb. Data sourced from Wang et al. [34]. [file 13227_2015_1_MOESM6_ESM.tiff]

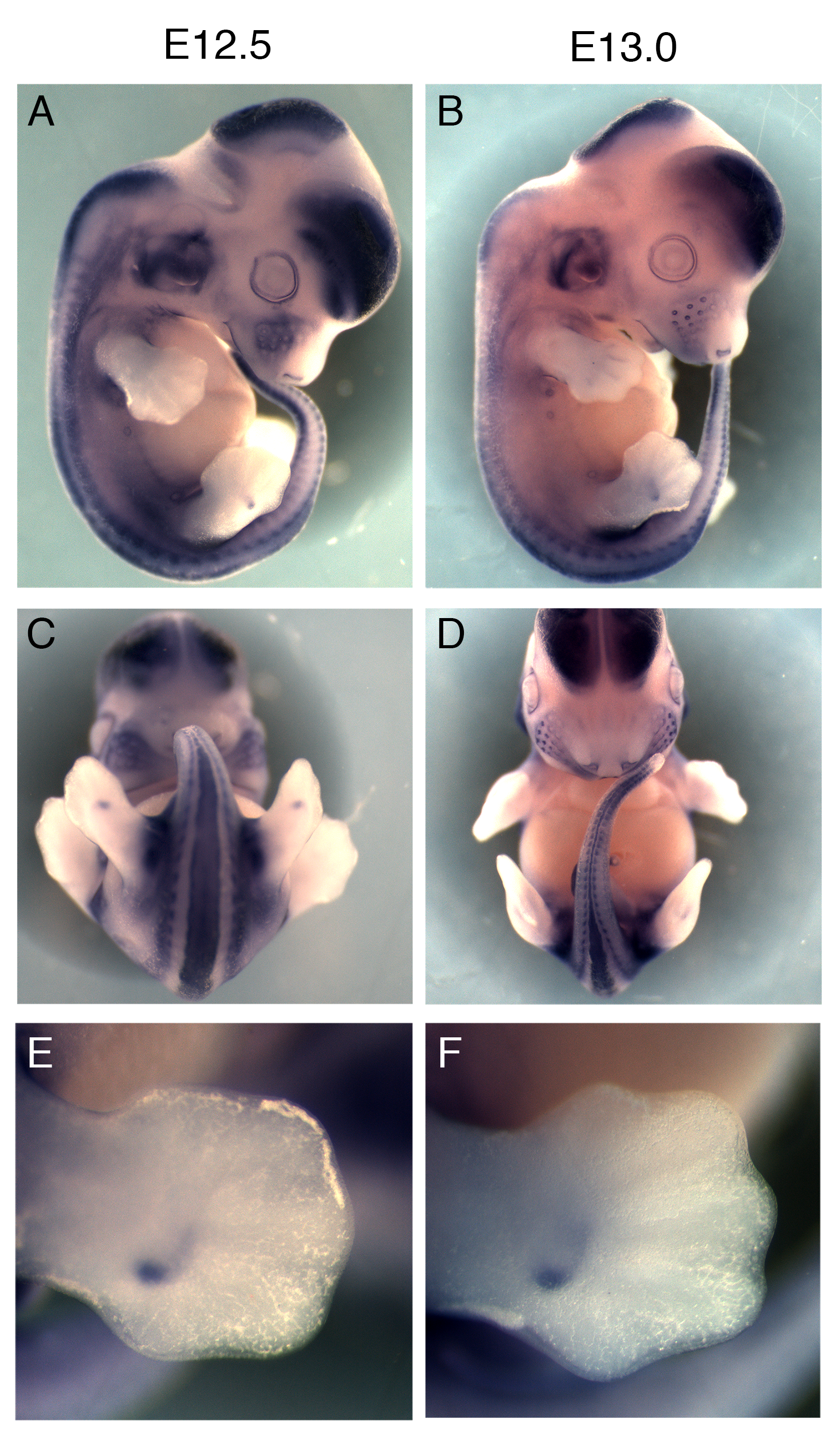

Supplement: Additional file 7: Figure S4. — The 3′-Meis in situ probe in the mouse HL detects a distinct ‘comma-shaped’ domain of expression during early autopod formation. Lateral (A, B), rostral (C) and ventral (D) views of whole E12.5 and E13.0 embryos show the full extent of 3′-Meis2 probe signal with the distinct footplate signal occurring in both left and right limbs (white arrows A, B and C). A high-magnification image of the footplate at E12.5 and E13.0 shows the full extent of this staining before it is lost at E13.5. [file 13227_2015_1_MOESM7_ESM.tiff]

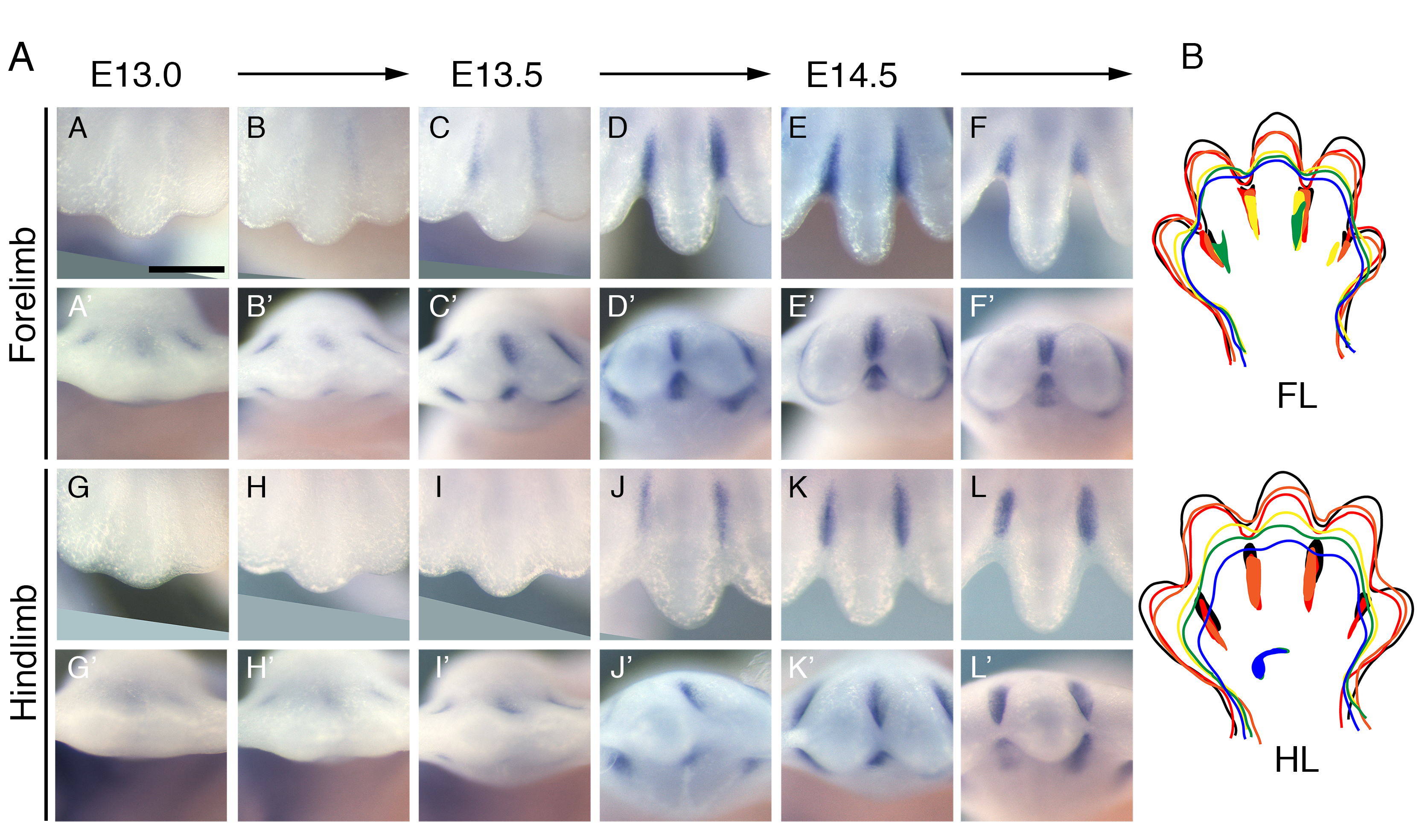

Supplement: Additional file 8: Figure S5. — Hybridisation of the 3′-Meis2 probe in the developing mouse autopod is limited to interdigital tissue that is retained. A time series of the expression in both the forelimb and the hindlimb indicates that Meis2 is expressed in both the dorsal and ventral surface of the autopod, in the region of the interdigits, over the period of interdigital thinning. A schematic of the staining over these stages gives an indication of the growth of the autopod and the regression of the interdigital tissue during this time. Dorsal views of autopods are shown. Scale bar represents 500 μm. [file 13227_2015_1_MOESM8_ESM.tiff]

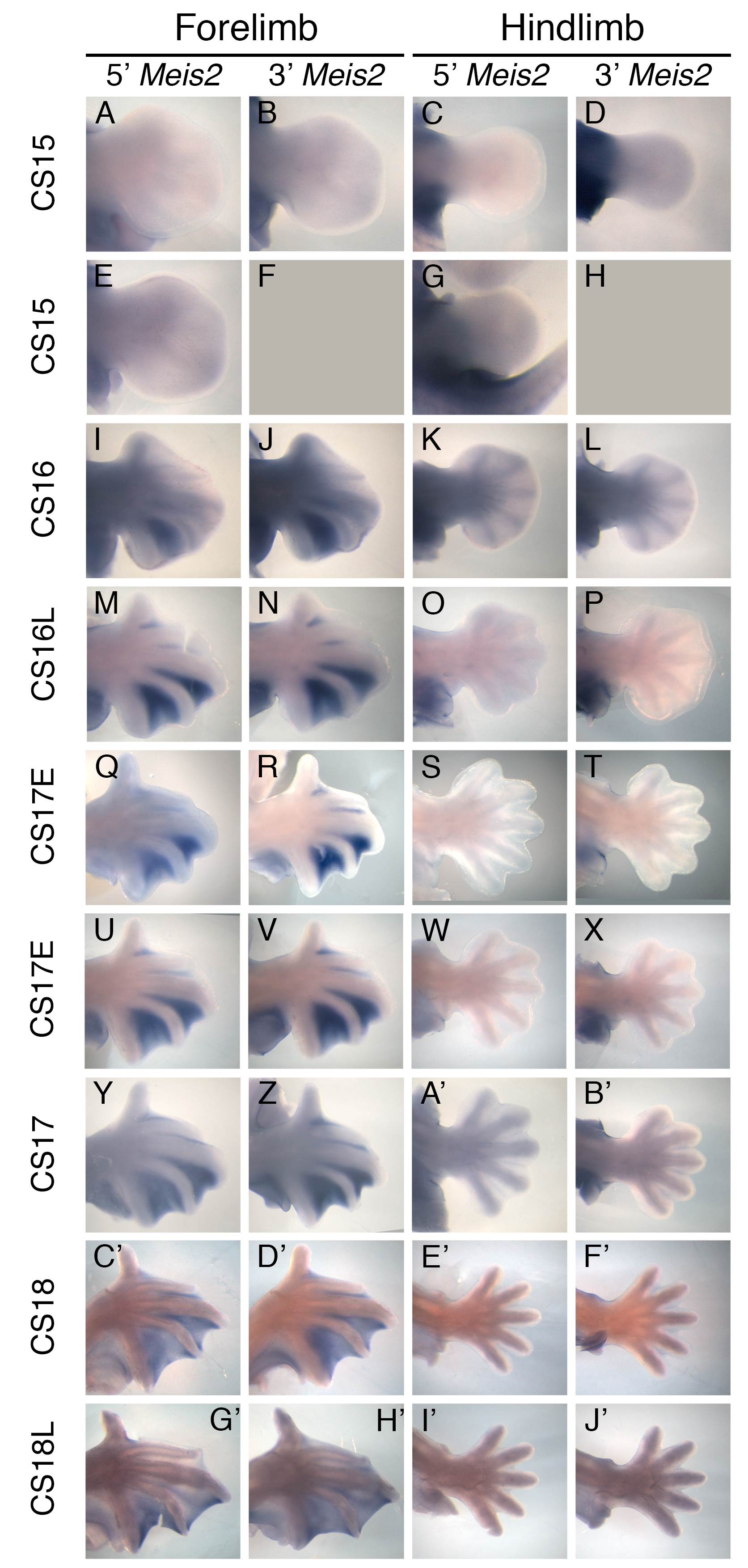

Supplement: Additional file 9: Figure S6. — Meis2 expression in developing bat limbs showing the biological repeats that were performed with both the 5′-Meis2 and the 3′-Meis2 in situ probes. Dorsal views of autopods are shown. Scale bars represent 500 μm. [file 13227_2015_1_MOESM9_ESM.tiff]

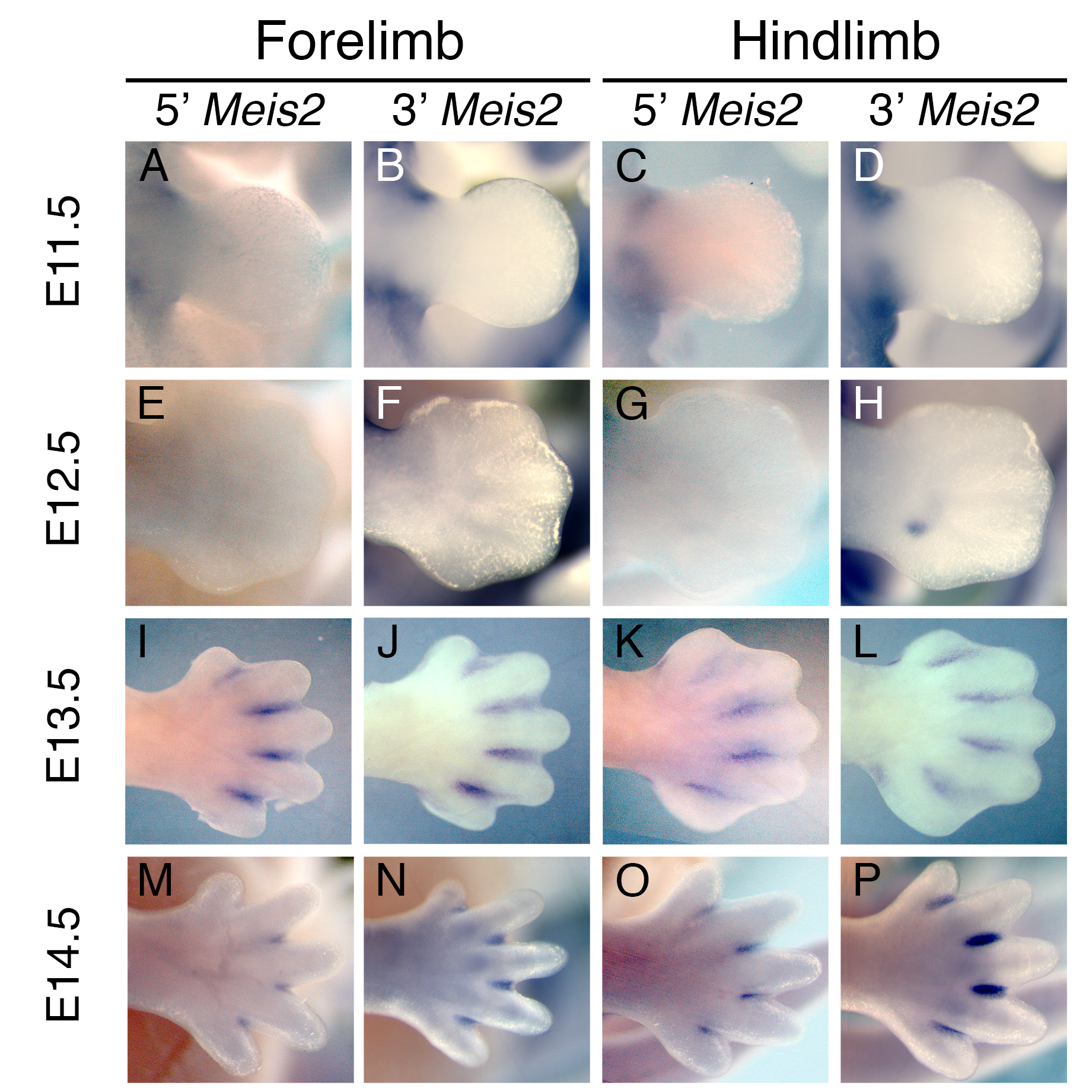

Supplement: Additional file 10: Figure S7. — Meis2 expression in developing mouse limbs indicates that there are few differences in signal between the 5′-Meis2 and the 3′-Meis2 in situ probes. Both probes show proximal expression in the developing FL and HL limb buds at E11.5 (A, B, C, D). The E12.5 autopods do not show any signal for the 5′-Meis2 probe (E, G) but a small comma-shaped domain of expression is seen in the HL with the 3′-Meis2 probe (H). However, during later stages of development (E13.5 and E14.5), both probes detect the same spatial expression pattern. Meis2 is expressed in the interdigital regions and is maintained in the proximal region of the interdigital tissue as regression occurs. Dorsal views of autopods are shown. [file 13227_2015_1_MOESM10_ESM.tiff]

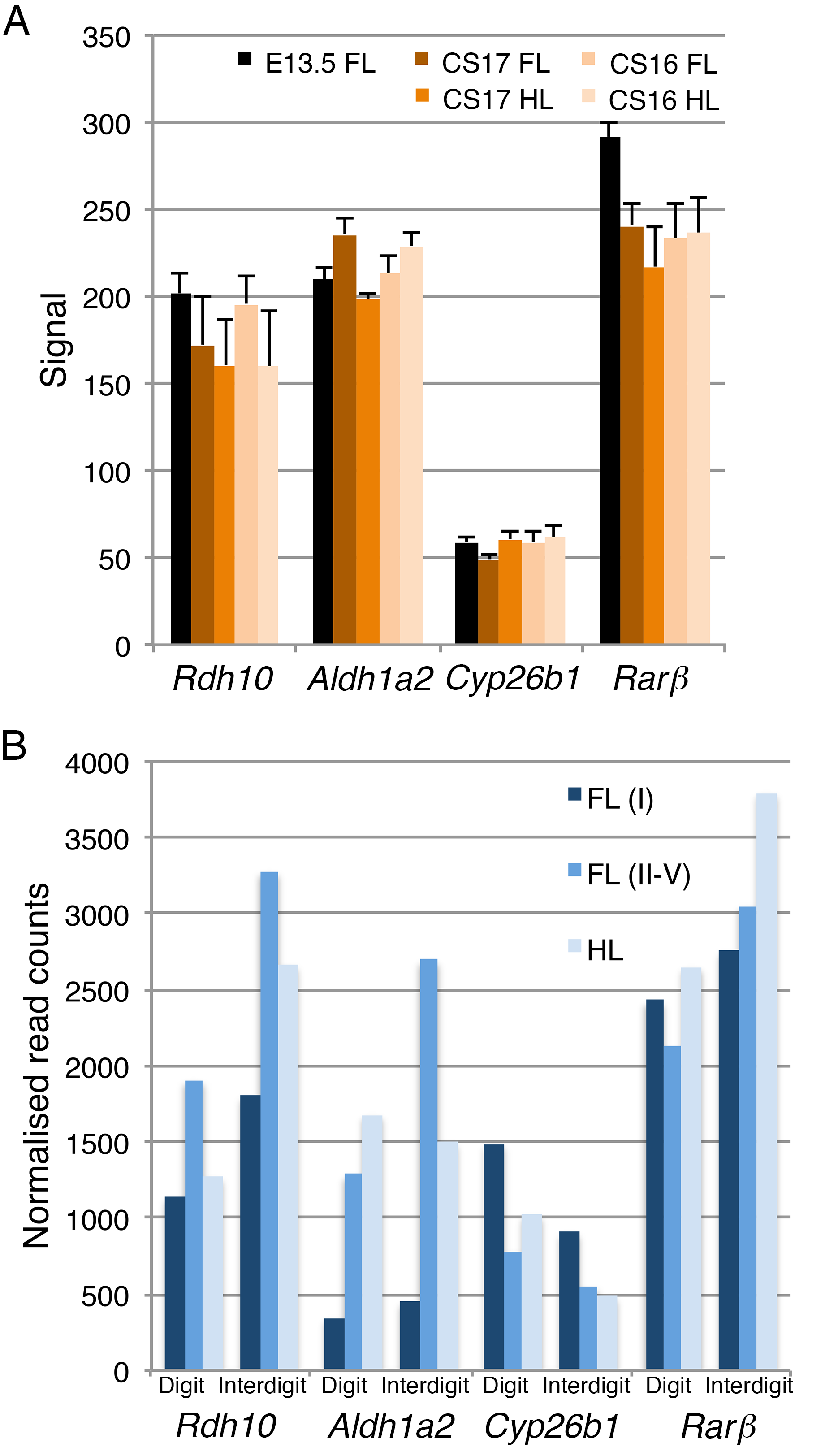

Supplement: Additional file 11: Figure S8. — RA synthesis, degradation and signalling data (A) The expression of genes involved in RA synthesis, degradation and signalling were not significantly differentially expressed in microarray analyses comparing E13.5 mouse FL, CS16 and CS17 M. natalensis FL and HL. (B) Analysis of RNA-seq data from pooled CS15-CS17 M. schreibersii FL and HL samples. The FL tissues were separated into the anterior portion (I) containing either digit I and the adjacent interdigit or the posterior portion (II to V) containing digits II to V and the corresponding interdigits. Data sourced from Wang et al. [34]. [file 13227_2015_1_MOESM11_ESM.tiff]
